# The Neuroimmune System and the Cerebellum

**DOI:** 10.1007/s12311-023-01624-3

**Published:** 2023-11-10

**Authors:** Donna L. Gruol

**Affiliations:** Neuroscience Department, The Scripps Research Institute, La Jolla, CA 92037 USA

**Keywords:** Microglia, Astrocytes, Cytokines, Chemokines, Activated glia, Glutamate homeostasis

## Abstract

The recognition that there is an innate immune system of the brain, referred to as the neuroimmune system, that preforms many functions comparable to that of the peripheral immune system is a relatively new concept and much is yet to be learned. The main cellular components of the neuroimmune system are the glial cells of the brain, primarily microglia and astrocytes. These cell types preform many functions through secretion of signaling factors initially known as immune factors but referred to as neuroimmune factors when produced by cells of the brain. The immune functions of glial cells play critical roles in the healthy brain to maintain homeostasis that is essential for normal brain function, to establish cytoarchitecture of the brain during development, and, in pathological conditions, to minimize the detrimental effects of disease and injury and promote repair of brain structure and function. However, dysregulation of this system can occur resulting in actions that exacerbate or perpetuate the detrimental effects of disease or injury. The neuroimmune system extends throughout all brain regions, but attention to the cerebellar system has lagged that of other brain regions and information is limited on this topic. This article is meant to provide a brief introduction to the cellular and molecular components of the brain immune system, its functions, and what is known about its role in the cerebellum. The majority of this information comes from studies of animal models and pathological conditions, where upregulation of the system facilitates investigation of its actions.

## Introduction to the neuroimmune system of the brain

It is well known that the peripheral immune system is a host defense system that protects the body against adverse conditions such as the presence of pathogens (e.g., bacteria and viruses), tissue damage, debris, toxic chemicals and other insults that cause disease or injury. A variety of cell types comprise and perform the basic functions of the peripheral immune system including leukocytes (e.g., macrophages, neutrophils, mast cells, eosinophils, basophils, dendritic cells) and lymphocytes, which are a special type of leukocyte (e.g., T cells, B cells and natural killer cells). Leukocytes and lymphocytes are produced from a multipotent cell type in the bone marrow known as hematopoietic stem cells. The immune cell types have specific functions that are critical to the successful operation of the peripheral immune system. Most peripheral immune cells are mobile and circulate in the blood and lymphatic vessels where they survey the body to locate sites of adverse conditions and insults. At such sites, immune cells, though the production and action of small proteins referred to as immune factors, co-ordinate and orchestrate complex multicellular behaviors involved in the recovery and repair programs that negate or minimize the effects of adverse conditions on the body.

Historically the brain has been considered an ‘immune privileged’ site, reflecting the capacity of the brain environment to limit the influence of the peripheral immune system on brain function, although peripheral immune cells do traffic through the brain looking for pathogens or evidence of other adverse conditions, and to interact with brain cells. However, recent research, much of it involving animal models of human diseases, has now established that the brain has its own innate immune system, called the neuroimmune system, which like the peripheral immune system, acts to protect the brain from detrimental consequences of adverse conditions (e.g., pathogens, disease, injury). Cells of the neuroimmune system produce and secrete some of the same immune factors as the peripheral immune system, but these factors are referred to as neuroimmune factors when produced by the cells of the brain. Importantly, the neuroimmune system also plays essential roles in the healthy brain, as a homeostatic regulator of physiological processes that maintain the brain in a balanced state (i.e., a homeostatic state), which is crucial for normal brain function, and as a regulator of brain development. However, if conditions cause the neuroimmune system to become dysregulated, its actions can contribute to the negative effects of the adverse conditions. For example, excessive production of neuroimmune factors is thought to be a contributing factor to the brain dysfunction characteristic of many pathological conditions such as neurodegenerative disease, psychiatric conditions, brain injury, and infection [50, 119, 120, 138].

**Fig. 1:**
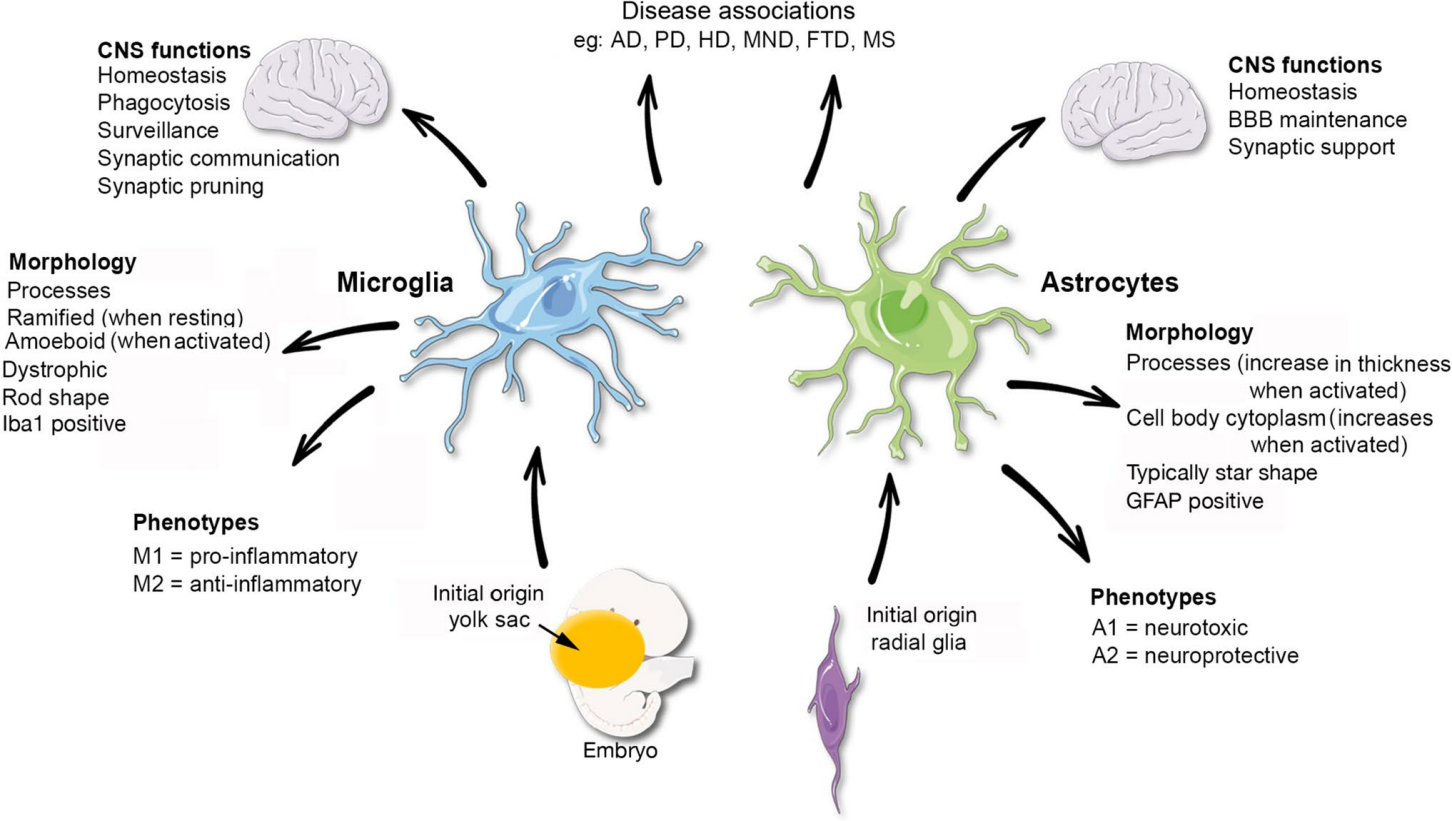
Overview of origin, function and morphological characteristics of microglia and astrocytes of the neuroimmune system. Abbreviations: AD, Alzheimer’s disease; PD, Parkinson’s disease; HD, Huntington’s disease; MND, motor neuron disease; FTD, frontotemporal dementia; MS, multiple sclerosis. (Reprinted with minor modification “Microglia and Astrocyte Function and Communication: What Do We Know in Humans?” by Garland, E, et al., 2022, Front Neurosci, p. 3 [41])

The recognition that glial cells of the brain, primarily microglia and astrocytes, function as an innate immune system of the brain evolved from early studies (1990’s) on the neurological consequences of human immunodeficiency virus (HIV) infection [40, 147]. In these early studies, it was found that HIV infected the brain shortly after peripheral infection and that the brain infection produced a variety of neurological symptoms such as cognitive disfunction, dementia, abnormal behavioral as well as motor dysfunction. These findings led to the discovery that the virus only rarely infected brain neurons, that glial cells of the brain, primarily microglia, harbored the virus, and that in response to viral infection microglia produced immune factors that altered the biology of brain cells, including microglia, astrocytes and neurons, resulting in altered brain function [40, 60, 88, 147]. It is now widely accepted that a neuroimmune system exists in the brain and plays critical roles in brain function under both physiological and pathological conditions. It is also widely accepted that: (a) astrocytes and microglia are the primary cell types that comprise the neuroimmune system, (b) both astrocytes and microglia have multifunctional roles, some of which overlap and involve cooperative actions, (c) astrocytes and microglia can accomplish many functions, comparable in certain respects to those performed by peripheral immune cells, and (d) the production and secretion of signaling factors classically known as immune factors, but referred to as neuroimmune factors when produced by brain cells, play an important role in the actions of the neuroimmune system on the brain [18, 21, 25, 41, 44, 56, 132, 133](Fig. 1).

The neuroimmune system extends throughout all brain regions, including the cerebellum. Studies of this systems is one of the fastest-growing fields today. However, attention to the cerebellar neuroimmune system has lagged that of other brain regions. For example, a PubMed search using the term ‘neuroimmune and cerebellum’ between the years 1992 and 2023 revealed 56 citations, compared to 396 citations for the term ‘neuroimmune and hippocampus’ and 348 citations for the term ‘neuroimmune and cortex’. This regional discrepancy may reflect the fact that the neuroimmune system has been primarily studied with respect to conditions that affected cognitive function such as occurs in neurodegenerative disorders, brain injury and viral or bacterial infection, whereas classically the cerebellum was thought to be involved primarily in motor control. However, it has now become clear that the cerebellum has an important impact on the functioning of many brain regions through reciprocal circuit connections and is involved in a wide range of behaviors including cognitive function [114, 115]. Moreover, emerging research has revealed an involvement of the neuroimmune system in several conditions associated with cerebellar motor dysfunction such as ataxias, a group of neurological disorders exemplified by loss of balance and coordinated motor function. For example, preclinical studies in mouse models have implicated a role for both microglia and astrocytes in spinocerebellar ataxias (SCA), diseases that are caused by the expansion of CAG trinucleotide repeats and are among the most understood ataxias [27, 35, 145]. Thus, understanding the structure and functioning of the cerebellar neuroimmune system is basic to understanding cerebellar function and mechanisms responsible for the healthy state and alterations that occur in disease and injury.

The neuroimmune system is very complex with many interacting cells, pathways and molecular players. It has become a focus of recent research and much of the information is new, with much more to be discovered, especially with respect to the cerebellum. As information on the cerebellum is limited, the goal of this article is to provide general information about the neuroimmune system of the brain and its physiological and pathological roles, with specific information on the cerebellum where available. The majority of this information comes from studies of animal models, particularly those involving disease or injury, and may differ somewhat from what is known about the neuroimmune system in the human brain, where studies are more limited in scope and primarily focus on pathological conditions. References, predominately review articles, are provided for some topics that can be referred to for further information. In addition to neuroimmune factors, cells of the neuroimmune system also produce factors such as nitric oxide (NO), reactive oxygen species (ROS), excitotoxins (e.g., glutamate) and complement proteins, and these factors can also contribute to the actions of neuroimmune cells depending on conditions but are beyond the scope of this article. Some of the cited references discuss these factors (e.g., [25]). Also beyond the scope of this article are interactions between the peripheral immune system and the central immune system, which can be particularly important in pathological conditions (e.g., [11, 62]).

## Neuroimmune factors and their receptors

### Immune factors

The primary immune factors produced and released by astrocytes and microglia are small (~ 6–70 kDa) signaling proteins that belong to a large superfamily of soluble proteins referred to as ‘cytokines’. These signaling factors play an important role in the actions of the neuroimmune system in both healthy and pathological states and are a focus of this article. The classification of a protein as a cytokine and its designation into a particular cytokine superfamily is generally based on similarities in structure and function. Within the cytokine superfamily there are superfamilies that differ structurally and functionally, although redundancy in function does occur, with several cytokines having the capability to subserve the same physiological, biochemical or pathological functions. Also, many cytokines are pleiotropic (e.g., interleukin-6) meaning that a single cytokine can elicit different biological responses often involving different cell types. The cytokine superfamilies include members of the chemokine, interleukin (IL), interferon (IFN), colony stimulating factor (CSF), transforming growth factor (TGF) and tumor necrosis factor (TNF) superfamilies [28]. Although chemokines are part of the cytokine superfamily, they are often referred to as a separate family rather than as a component of the cytokine superfamily (i.e., the term ‘cytokines and chemokines’ is used rather than the term ‘cytokine’).

Families exist within the superfamilies. The IL-1 family consists of 11 cytokines, IL-1α, IL-1β, IL-18, IL-33, IL-36α, IL-36β, IL-36γ, IL-36ra, IL-37, and IL-38. The IL-6 family consist of IL-6, IL-11, IL-27, ciliary neurotrophic factor (CNTF), leukemia inhibitory factor (LIF), oncostatin M (OSM), cardiotrophin 1 (CT-1), and cardiotrophin-like cytokine (CLC). The TNF superfamily contains 19 family members including TNFα, TNF-β, and TNFγ. The IFN superfamily consists of more than 20 members that are typically classified as Type I IFN, Type II IFN, or Type III IFN; within each class there are numerous members. Three key interferons are INFα, IFN-β, and IFNγ. Four members comprise the CSF family, G-CSF (granulocyte colony-stimulating factor), M-CSF (CSF1; macrophage colony-stimulating factor), GM-CSF (CSF2; granulocyte–macrophage colony-stimulating factor), and IL-3. The TGF-β family consists of 33 members.

In spite of the large number of cytokines that have been identified, only a relatively small percentage have been detected in the brain, perhaps because the neuroimmune system is an emerging field and information is still limited. Technical issues concerning the detection of low levels of proteins could also be a contributing factor. Moreover, the neuroimmune system may not use all the immune factors that are used by the peripheral immune system, a possibility that will take further scientific inquiry to resolve. Some of the major cytokines shown to be expressed in the cerebellum at the mRNA or protein levels under normal or pathological conditions include IL-1β, IL-6, IL-9, IL-10, IL-15, IL-8, TNFa, CCL2, CXCL12, CXCL14, IP-10, MIP1α, GM-CSF and TGFβ.

Levels of neuroimmune factors in the brain are tightly regulated under normal homeostatic conditions (i.e., physiological conditions) and are difficult to detect, due to the low levels (pM or lower) at which they are produced and secreted or other issues such as the dynamic nature of their secretion processes and their short half-lives. Thus, studies of the neuroimmune system have primarily involved adverse or pathological conditions when increased production (e.g., nM or higher levels) occurs as part of the defense, recovery, and repair functions of astrocytes and microglia. Typically, levels of neuroimmune factors in the brain are measured by immunoassays such as enzyme-linked immunoassay (ELISAs), which can determine the level of a single protein, or multiplex analysis systems that can simultaneously determine levels of multiple cytokines [73]. Measurement of neuroimmune factors at mRNA level is also common and can detect low levels of mRNA. However, although detectible expression occurs at the mRNA level, it may not be translated to expression at the protein level. Nevertheless, the presence of mRNA suggests the ability of the cell to express the protein when conditions call for it. A number of preclinical or clinical studies have demonstrated the expression of cytokines/chemokines in the cerebellum of animal models of pathological conditions or in human patients, suggesting a role for these factors in the pathological condition [106]. Some examples of these studies are listed in Table 1. Note that the cellular source of the factor was not always identified and could have involved a contribution from peripheral immune cells (e.g., infiltrating macrophages and T cells) trafficking through the cerebellum in addition to cerebellar glial cells.

**Table 1:** Representative examples of preclinical and clinical studies showing altered expression of neuroimmune factors in the cerebellum

Condition	Neuroimmune factors	Source cell type	Species	Reference
lead exposure	IL-1β, IL-6,TGFβ	nd^1^	rat pups	[20]
hypoxic neonatal rat brain	TNFα, and IL-1β	activated microglia	rat pups	[61]
asphyxia	TNFα, IL-10, IL-1β, IL-6	nd	fetal rat	[140]
hepatic encephalopathy	IL-1β and TNFα, IL-4	microglia and astrocyte activation	rats	[1]
mercury-induced deficits in social behavior	TNFα	Glial activation	male voles	[127]
wobbler mouse, as a model for Amyotrophic lateral sclerosis (ALS)	IL-1β, TNFα1, TGF-β1, IL-10	microglia and astrocyte activation	mouse	[109]
Spinocerebellar Ataxia Type 1 mouse models	TNFα, IL-6, MCP-1	microglia and astrocyte activation	mouse	[27]
Sporadic Creutzfeldt–Jakob disease (sCJD) MM1 and VV2 subtypes	TGFβ1, TGFβ2, IL10, IL10Rβ, IL6st, IL8, TNFα, TNFRSF1A,	mainly microglia, IL6, IL10RA, and TNF-α in glial cells, IL10, M-CSF, and TNFα in neurons	postmortem cerebellar tissue from sCJD patients	[74]
Fragile X Tremor and Ataxia Syndrome (FXTAS)	IL-12,TNFα	nd	postmortem cerebellar tissue from FXTAS cases and controls	[30]
Autism	MCP-1 and IL-6	astrocytes	postmortem cerebellar tissue from autistic cases and controls	[135]
Lurcher mouse, model for ataxia	IL-1β	nd	mouse	[139]
Rat model of pediatric mild traumatic brain injury	CXCL1, IL-5, VEGFα	nd	rat	[37]
Mouse model of lupus	mRNA for IL-6, IFNγ, IL-1β, and IL-10	nd	mouse	[129]
Mouse model of Alzheimer’s disease	TNFα, MCP-1	nd	mouse	[64]
Alcohol (ethanol)	mRNA analysis of IL-1β, TNF-α, CCL2, IL-18, TGFβR1	nd	mouse	[86]
Alcohol (ethanol)	CCL2 and IL-6 in the adult but not the adolescent cerebellum	nd	mouse	[59]
Viral infection	IL-1α,IL-1- β, IL-6 TNFα	reactive astrocytes and activated microglia	rats	[111]

*nd*^*1*^ not determined

Cytokines and chemokines are commonly described as acting in a proinflammatory or anti-inflammatory manner, terms that reflect functional roles. Inflammation, which is commonly referred to as neuroinflammation when it occurs in the brain, is a local response to cell injury or infection that is designed to stop the negative effects of an insult and promote healing and repair. Proinflammatory cytokines promote the inflammatory reaction to stop the negative effects of detrimental conditions but can make conditions worse if the actions are uncontrolled. Anti-inflammatory cytokines act in opposition to the proinflammatory cytokines and serve to reduce inflammation and promote healing and repair. The cytokines TNF-α, IL-6, IL-1β, IL-12, and CCL2 are considered the major proinflammatory cytokines in the brain, whereas the cytokines interleukin-1 receptor antagonist (IL-1Ra), IL-4, IL-10, IL-11, and IL-13 are considered the major anti-inflammatory cytokines. However, context plays an important role in the functional consequences of cytokine action. Issues such as the level of expression of the cytokine, cellular source, target cell type, receptors expressed by target cells and signaling pathway activated, as well as the presence of other interacting factors can be deciding factors in whether the cytokine is acting in a pro-inflammatory or anti-inflammatory manner. For example, IL-6 is considered a proinflammatory cytokine, but it can also have beneficial effects reflective of anti-inflammatory actions [39, 113]. Under healthy conditions, proinflammatory and anti-inflammatory neuroimmune factors act in concert to perform a multitude of roles that are essential for the normal development and function of the brain.

Inflammation can be initiated by the presence of a pathogen but can also occur when there is no pathogen present, for example when cell damage or brain injury occurs. When inflammation occurs in the absence of a pathogen, it is referred to as a sterile inflammation [7]. A sterile inflammatory response is initiated by endogenous molecules that are released from dead or necrotic cells or are modified during a disease process (e.g., misfolded or aggregated peptides such as occurs in Alzheimer’s disease). These molecules are referred to as damage-associated molecular patterns (DAMPs). Pathogen-associated molecular patterns also occur, are associated with inflammation initiated by a pathogen, and are referred to as PAMPs. Specialized pattern-recognition receptors (PRRs) on the surface of glial cells can recognize and bind to DAMPS and PAMPs resulting in PRR activation that initiates both biochemical and morphological changes associated with an activation phenotype of the glial cells. Depending on conditions, the activated glial cells then produce and secrete anti-inflammatory cytokines that act in a beneficial manner or proinflammatory cytokines that may contribute to further cellular damage [7]. Chronic activation of glial cells and over-production of neuroimmune factors is a common feature of neurodegenerative diseases and can lead to detrimental effects on brain structure and function [120].

**Fig. 2:**
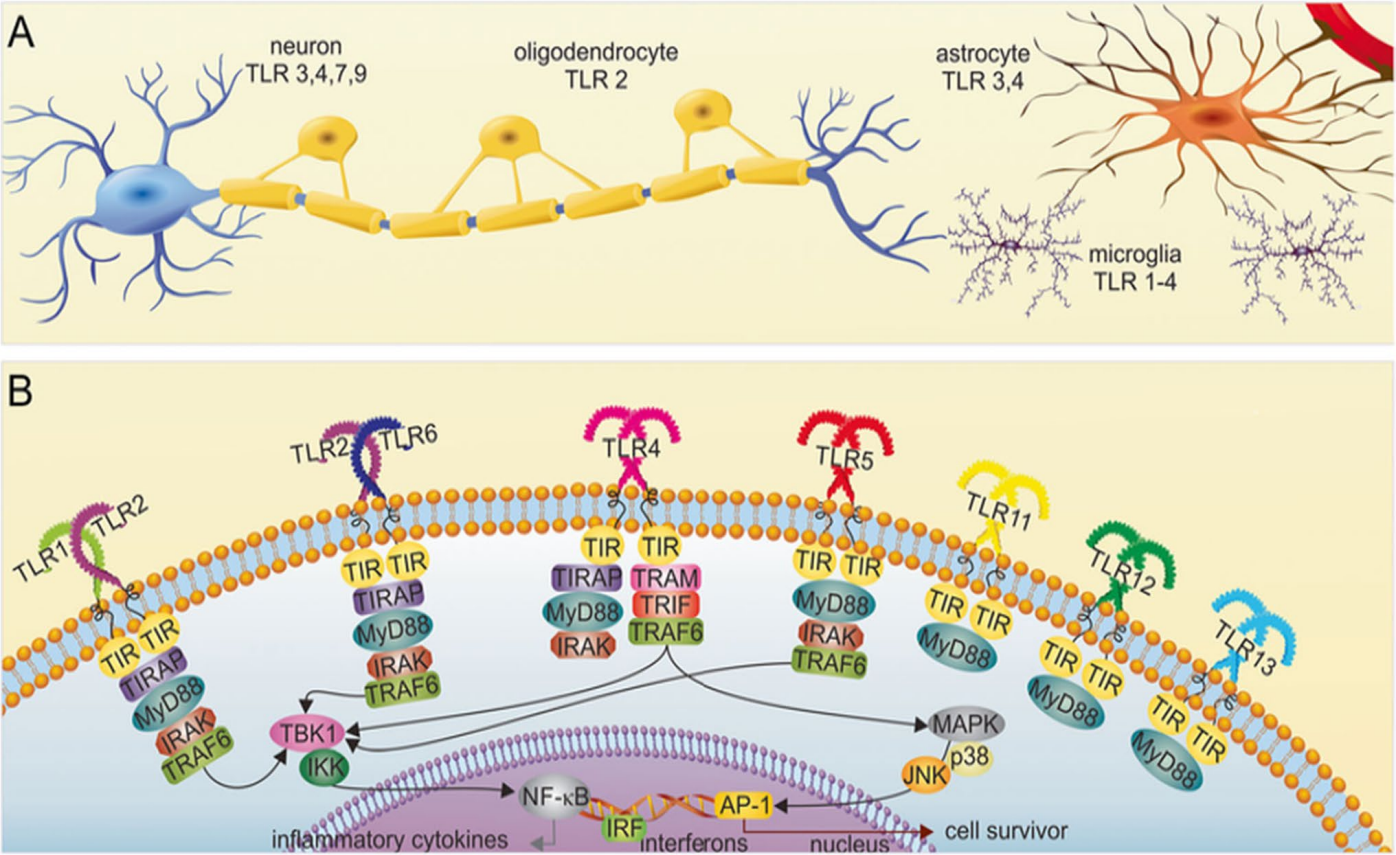
Toll-like receptors in the brain. A,B. Diagram illustrating toll-like receptor (TLR) expression that has been reported for neurons and glial cells of the brain (**A**) and the signaling partners associated with the receptor (**B**). Abbreviations: TIR, toll-interleukin-1 receptor domain; TIRAP, TIR-domain containing adapter protein; MyD88, myeloid-differentiation primary response gene 88; IRAK, interleukin-1 receptor associated kinase; TRAF, TNF receptor-associated factor (TRAF)-6 adapters; TANK, TRAF-family member-associated nuclear factor-ΚB (NF-κβ) activator; TBK-1, TANK-binding kinase-1; NF-κβ, nuclear factor kappa β; IRAK, IL-1 receptor (IL-1R)–associated kinase; TRAM, translocating chain-associated membrane protein; TRIF, TIR-domain-containing adapter-inducing interferon-β; MAPK, map kinase; JNK, c-Jun N-terminal kinase; AP-1, activator protein 1. (Reprinted from “Interplay Between Exosomes, microRNAs and Toll-Like Receptors in Brain Disorders” by Paschon, V. et al., 2016, Mol Neurobiol 53, p. 2020 [96])

Experimentally, one of the most commonly used methods to induce inflammation and production of neuroimmune factors is by the application of lipopolysaccharide (LPS), a bacterial toxin that is a major constituent of the outer membrane of gram-negative bacteria. LPS acts at PPRs. One of the best characterized families of PPRs are the toll-like receptors (TLRs), one of which (TL4) is activated by LPS and other PAMPs and DAMPs. TLRs are expressed on the cell surface (TLR1, 2, 4, 5, and 6) or in endosomal vesicles (TLR3, 7, 8, and 9) and signal through complex intracellular pathways that lead to gene expression (Fig. 2). In the brain, microglia and astrocytes are the primary cell types that express these receptors, but the receptors also can be expressed by other brain cells (e.g., oligodendrocytes, neurons, endothelial cells). Toll-like receptors in microglia have been intensively studied but information about toll-like receptors in astrocytes is relatively limited. Toll-like receptors play important roles in both normal and pathological conditions [69, 89].

### Receptors for immune factors

**Fig. 3:**
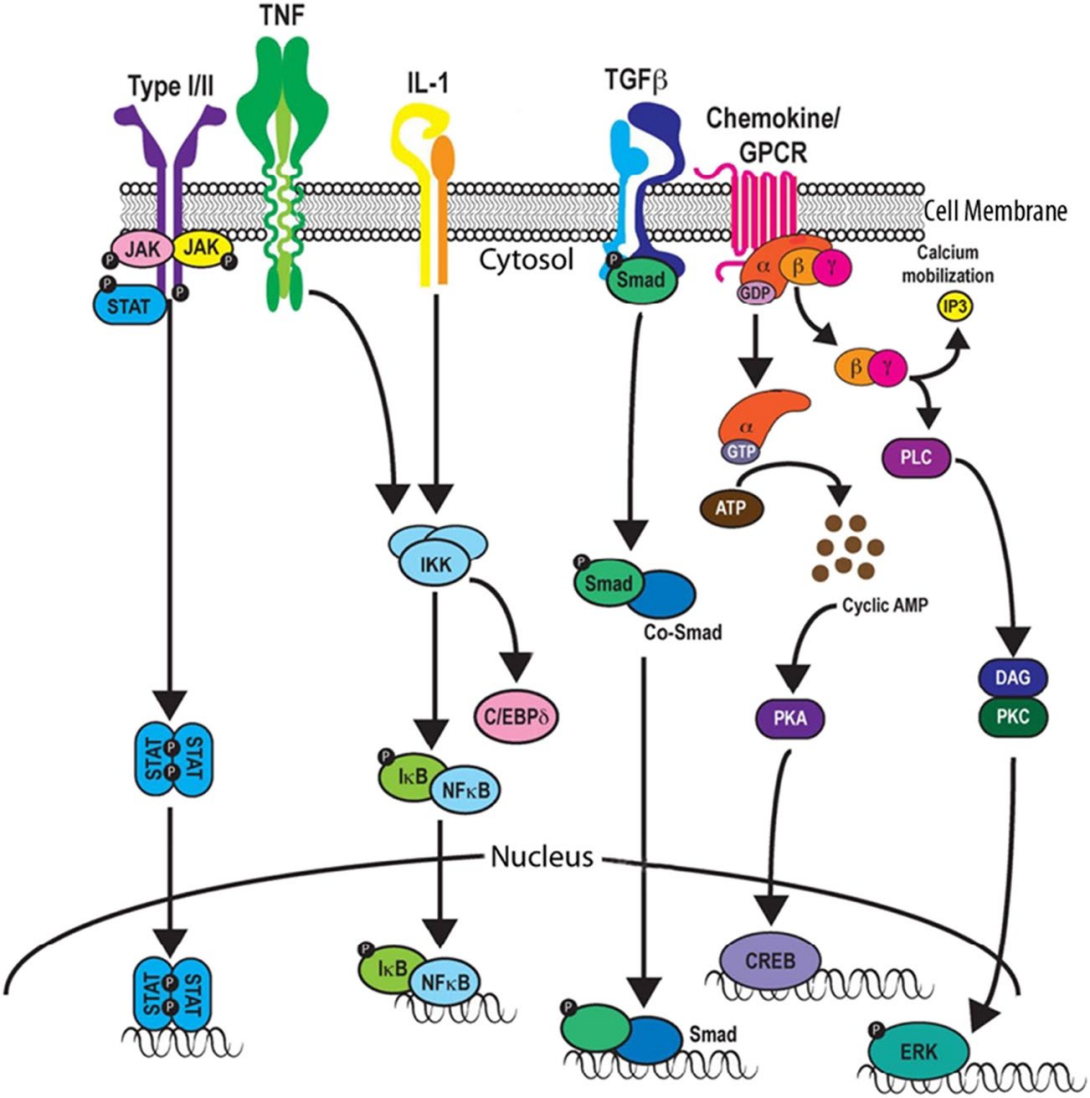
Cytokine receptors and signal transduction pathways that are activated by binding of the cytokine to the receptor. Abbreviations: JAK, Janus kinase; STAT, signal transducer and activator of transcription; TNF, tumor necrosis factor; IKK, Inhibitory-κβ Kinase; Iκβ, Nuclear Factor-κβ; NFκβ, Nuclear Factor-κβ; C*/*EBP delta, CCAAT/enhancer binding protein delta; Smad, mothers against decapentaplegic; CoSmad (Smad4), “common-mediator”; GPCR, G protein-coupled receptor; GDP, guanosine diphosphate; guanosine GTP, triphosphate; ATP, adenosine triphosphate; AMP, adenosine monophosphate; PKA, protein kinase A; CREB, cAMP response element-binding protein; IP3, inositol 1,4,5-triphosphate; PLC, Phospholipase C; DAG, diacylglycerol; PKC, protein kinase C: ERK, extracellular signal-regulated kinase.(Reprinted with modification from “Type I/II cytokines, JAKs, and new strategies for treating autoimmune diseases” by Schwartz, D. et al., 2016, Nat Rev Rheumatol 12, p. 19 [116])

Biological actions of neuroimmune factors are mediated by complex receptor systems that are classified into superfamilies based on similarities in structure and composition of subunits that form the receptors. For example, most of the interleukins utilize receptors classified as Type I receptors, INF and IL-10 utilize receptors classified as Type II receptors, and TNF utilizes the TNF family of receptors. Cytokine receptors generally consist of two to four receptor chains (i.e., subunits) that can be structurally distinct or identical with individual chains shared by members of the same receptor family to form distinct receptors. One or more of the receptor chains contain a cytokine (i.e., ligand) binding site and/or sites that interact with signal transduction partners. For example, generally Type 1 receptors are composed of two subunits, a unique a chain that contains the ligand binding/recognition site that confers specificity for a particular cytokine(s) and a common subunit that is utilized by all members of the particular cytokine family. The IL-1 family of receptors (IL-1R) is a Type 1 receptor family and is comprised of five ligand-binding subunits (IL-1R1, IL-1R2, IL-1R4, IL-1R5, and IL-1R6) and two types of accessory subunits (IL-1R3, IL-1R7). Receptors that only bind to a specific cytokine are referred to as a cognate receptor for that cytokine and are generally named according to the cytokine that binds to the receptor with the addition to the letter ‘R’ to designate receptor. A simplified diagram of several cytokine receptors and associated signal transduction pathways that lead to gene expression are illustrated in Fig. 3.

**Fig. 4:**
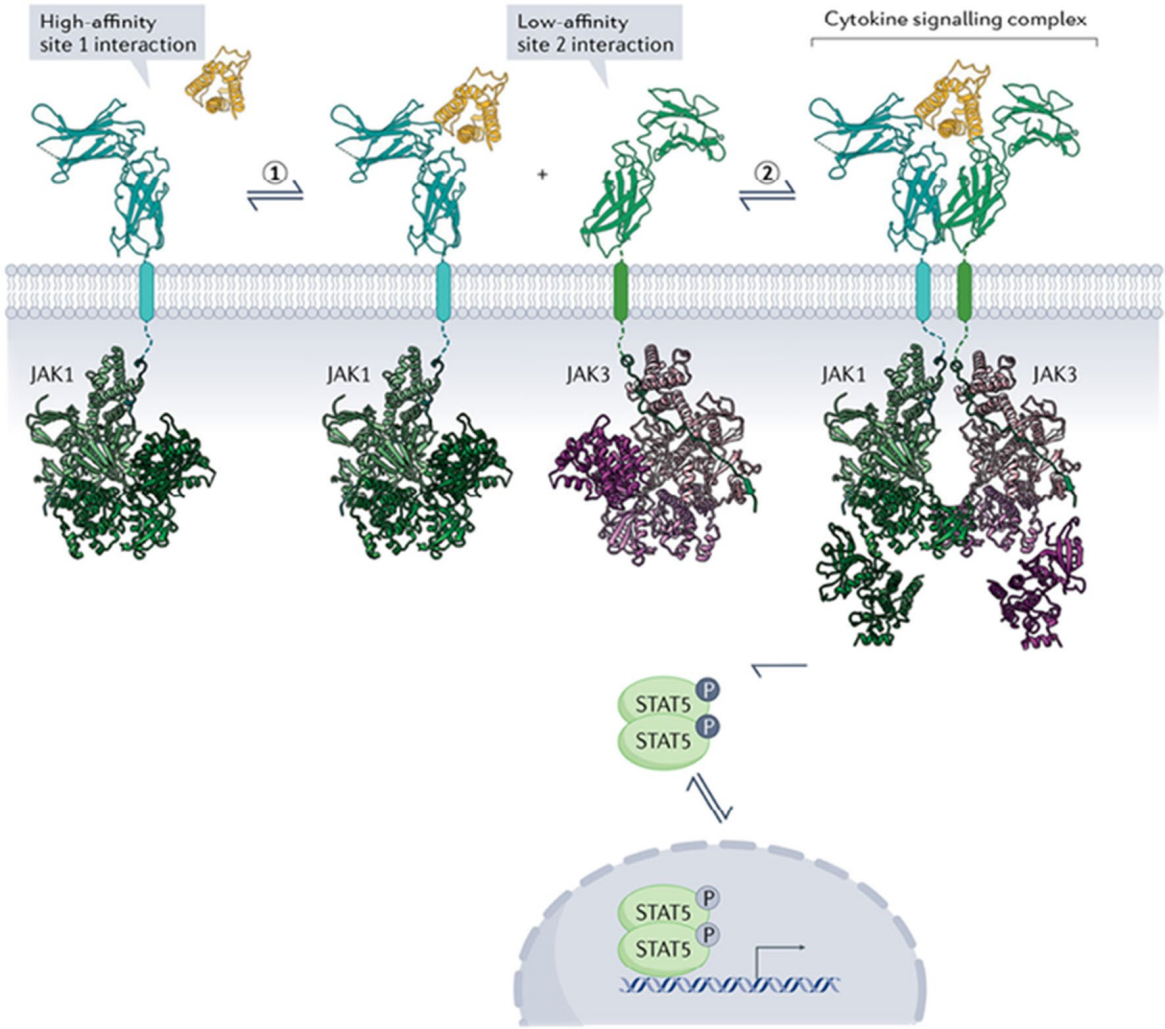
General model of cytokine signaling. Two-step activation is common among cytokine receptors. The cytokine (yellow) in the extracellular fluid first binds to site 1 of a high-affinity receptor subunit (cyan) of the cognate receptor located in the cellular membrane, which results in recruitment of the low-affinity membrane bound receptor subunit (green) to form a ternary cytokine receptor complex. The formation of the complex enables the activation and transphosphorylation of the intracellular Janus kinases (JAKs) (green, purple), triggering the phosphorylation STAT (a transcription factor) which then translocates to the nucleus where it regulates gene expression. (Reprinted with modifications from “Emerging Principles of Cytokine Pharmacology and Therapeutics” by from Saxon, R. et al., Nat Rev Drug Discov, 2023, 22, p. 25 [112])

Once a cytokine binds to the binding/recognition subunit (s) of a receptor, transduction of the cytokine signal occurs and involves an interaction of the binding/recognition subunit with one or more secondary subunits that express sites that interact with and activate specific intracellular signal transduction pathways (Fig. 4). For example, the IL-6 receptor family receptors link to non-receptor tyrosine kinase pathways and the transforming growth factor-β (TGF-β) family receptors link to the Smad signaling pathway. TNF in the brain primarily utilizes the TNF receptor 1 (TNFR1), which signals through nuclear factor-kappa β (NF-κβ) and mitogen-activated protein kinase (MAPK) pathways, but also has a death domain in its cytoplasmic part that can link to cytotoxic signal pathways that cause cell death. The final biological effect associated with receptor activation generally involves regulation of gene expression. However, the signal transduction components comprising a pathway can also regulate other biochemical/physiological processes, a situation that can make identification of mechanisms mediating the actions of neuroimmune factors challenging.

In addition to membrane bound receptors, some cytokines can also act through soluble receptors, which are formed by a variety of mechanisms including shedding from the membrane by proteolytic cleavage, alternative splicing of mRNA transcripts, or transcription of genes that encode a soluble form of a receptor [68]. Soluble receptors can regulate cytokine actions by a variety of mechanisms such as acting as agonists and activating signaling as is the case for the IL-6 soluble receptor (sIL-6Rα) or acting as antagonists that binds to the soluble receptor but cannot activate it and thus competes with membrane bound receptors for the cytokine, as is the case for the IL-1 soluble receptor (sIL-1Ra). A variety of cytokine receptors can form soluble receptors and multiple soluble receptors can exist for a particular cytokine with different mechanisms of action and endpoints [49, 75, 92].

**Fig. 5:**
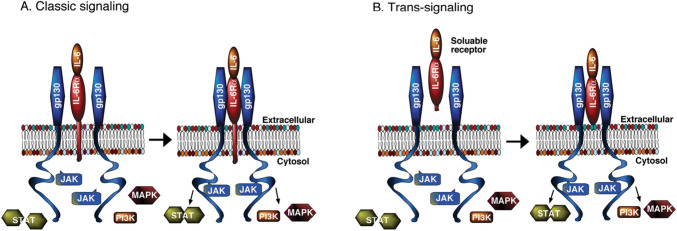
IL-6 signaling. IL-6 signal transduction can occur through two pathways, through a membrane bound receptor (classic signaling) or a soluble receptor (trans-signaling). The IL-6/IL-6R complex interacts with two membrane bound gp130 subunits which enables activation of a JAK/STAT signaling pathway. In addition, the IL-6/IL-6R/gp130 complex can activate RAS/mitogen-activated protein kinase (p44/42 MAPK, also called ERK1/2; MAPK) and phosphatidylinositol-3 kinase (PI3K) signaling pathways. All three signaling pathways activate downstream signaling molecules and effectors. (Reprinted from “Impact of Increased Astrocyte Expression of IL-6, CCL2 or CXCL10 in Transgenic Mice on Hippocampal Synaptic Function” by Gruol, D., 2016, Brain Sci, 6, p.3 [46])

An interesting and important aspect of IL-6 signaling is that the IL-6 receptor (IL-6R) itself does not have an intrinsic signaling element. Instead, the receptor utilizes a ubiquitous signaling subunit called glycoprotein 130 (gp130). All members of the IL-6 superfamily of ligands signal through a homo- or heterodimer of gp130. In the classical signal pathway, IL-6 binds to the membrane bound IL-6R enabling the formation of a trimer consisting of IL-6, IL-6R and two gp130s, which is the active receptor complex. The IL-6 receptor can also exist as a soluble receptor that can bind to IL-6 in the extracellular fluid. The soluble IL-6R/IL-6 complex is capable of binding to membrane bound gp130 resulting in an active receptor that can induce IL-6 signal transduction (Fig. 5). The ubiquitous distribution of gp130 in cells enables brain cells that do not endogenously express IL-6R to form active receptors that initiate signal transduction and through downstream actions contribute to the effects of IL-6 in the brain [107]. This form of IL-6 signaling is referred to trans-signaling and has received considerable attention with respect to the mechanisms mediating effects of IL-6, particularly under pathological conditions [108].

In contrast to Type I/II cytokines and receptors, members of the chemokine superfamily of cytokines utilize receptors with a seven transmembrane region that links to pertussis toxin sensitive G_αi_-protein, which couples to signal transduction pathways (see Fig. 3). Chemokines are chemotactic cytokines that can induce directed movement (i.e., chemotaxis) in cells responsive to a chemical gradient of a specific cytokine(s). Chemokines are particularly important during cerebellar development where they direct cell movement to form the appropriate cerebellar architecture. Chemokines are classified into four categories as CXC, CC, C and C_3_C based on the placement of conserved cysteine residues within the peptide structure, and the receptors are classified according to the CXC, CC, C or CX3C ligand that binds the receptor, however some chemokine receptors are promiscuous and can bind chemokines from more than one ligand class, as shown in Table 2.

**Table 2:**
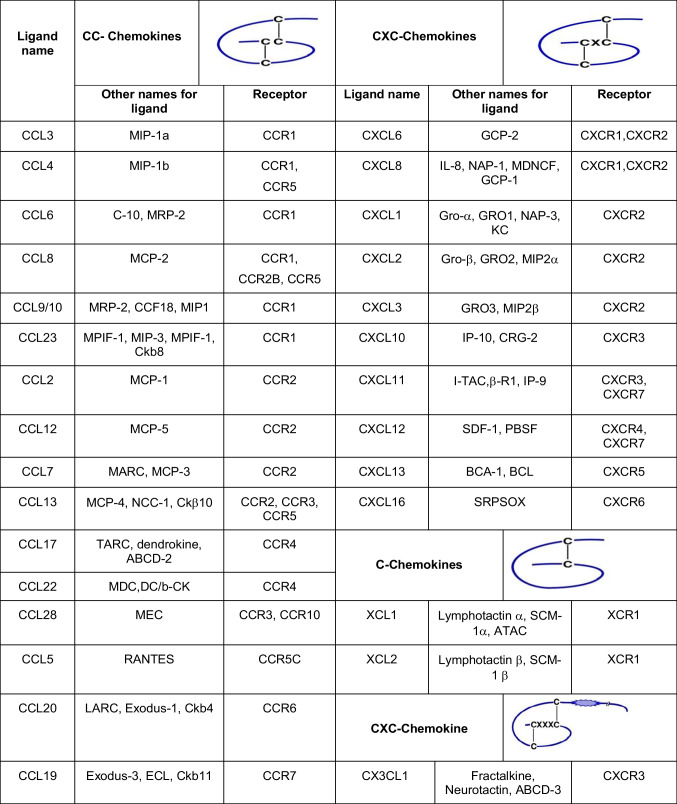
Representative examples of chemokines and their receptors

The expression of specific cytokine receptors in the brain varies across cell types and brain regions. However, microglia and astrocytes express many of the same cytokine receptors, secrete the same cytokines, and are activated by the same cytokines. Thus, cytokines produced and secreted by microglia or/and astrocytes can alter the function of the producing cells and/or other microglia and astrocytes present in the local environment. Neurons also express receptors for cytokines and neuronal function can also be impacted by the factors produced by local microglia and astrocytes. Environmental conditions, for example the concentration of a cytokine or the presence of a cell type specific stimulant, are important determinates of the cell type that first initiates cytokine secretion, which then influences activities of other cell types in the local environment that express the cognate receptors. A number of studies under healthy or pathological conditions have shown that the cerebellum expresses receptors for many neuroimmune factors. For example, several chemokine receptors are expressed in the cerebellum including CXCR1, CXCR2, CXCR3, CXCR4 [76, 103]. Receptors for IL-6 and LIF are expressed on Purkinje neurons [79]. TNF1 receptors for TNF are expressed in Bergman glia [117].

### Actions of neuroimmune factors in the cerebellum

A variety approaches and models have been used to gain an understanding of the function of the neuroimmune system and the effects of neuroimmune factors on brain cells, although only a limited number of studies have focused on cerebellar cells. This section summarizes some of the studies that have appeared involving mature or developing cerebellar cells. Taken together, the studies show that neuroimmune factors have a diversity of effects on mature and developing cerebellar cells under both physiological and pathological conditions, consistent with important roles for the cerebellar neuroimmune system.

Of the neurons that comprise the cerebellar cortex of adult mammals (Purkinje cells, Golgi cells, granule cells, basket cells, stellate cells, Lugaro cells, unipolar brush cells, candelabrum cells), Purkinje neurons have been a favored neuron for study because of their large size, accessibility and importance to cerebellar function, as they provide the only output from the cerebellar cortex, and thus, play a critical role in the transmission of information from the cerebellum to the rest of the brain. A variety of studies using exogenous application of neuroimmune factors in rodent models (in vivo or ex vivo) have shown that neuroimmune factors can influence the activity of Purkinje neurons by altering the firing rate or synaptic transmission. For example, in vivo electrophysiological recordings of spike firing activity of Purkinje neurons in anesthetized mice showed that focal administration of IL-1β to the Purkinje neurons, which have been shown to express IL-1β receptors [38], increased the spike firing rates of the Purkinje neurons [80]. Also, local microinjection of IL-1β into the cerebellum in vivo produced ataxia, whereas local microinjection of IL-6 did not, suggesting a role for IL-1β in ataxia [2].

In electrophysiological studies of excitatory synaptic events (derived from either parallel fibers or climbing fibers; source not identified) recorded in Purkinje neurons in slice preparations of mouse cerebellum, application of IL-1β enhanced the excitatory synaptic events, while application of TNFα had no effect [77]. Associated with the enhancement of synaptic events was a downregulation by IL-1β of the glutamate-aspartate transporter/excitatory amino acid transporter 1 (GLAST/EAAT1), a glial protein that is responsible for uptake of extracellular glutamate during excitatory synaptic transmission [77]. These results suggest that IL-1β by acting on glial cells can regulate synaptic transmission at excitatory synapses to the Purkinje neurons.

Using a similar approach, TNFα applied to rat Purkinje neurons in cerebellar slices in vitro increased spike firing of the Purkinje neurons and produce a prolonged increase in excitability [117]. Results from additional studies identified the underling mechanism as involving an action of TNFα to evoke an increase in glutamate release from the Bergman glia cells, which are closely associated with Purkinje cell dendrites, resulting in glutamate activation of metabotropic glutamate receptors (mGluR) on the Purkinje neuron dendrites [117]. The activated mGluRs increased excitability of the Purkinje neurons through a G-protein coupled signal transduction pathway that regulates the function of ion channels responsible for excitability. This mechanism contrasts to mechanisms responsible for changes in excitability produced by activation of other subtypes of glutamate receptors (e.g., NMDARs (N-methyl-D-aspartate receptors) and AMPAR (α-amino-3-hydroxy-5-methyl-4-isoxazolepropionic acid receptors). For these receptors, the ion channel is part of the receptor complex and ligand binding to the receptor directly links to channel activation.

Chemokines are also effective in altering synaptic transmission in Purkinje neurons. Application of the CXC chemokine interleukin-8 (IL-8) or growth-related gene product α (GROα) to Purkinje neurons in mouse cerebellar slices increased the number of spontaneous inhibitory and excitatory synaptic events and increased the amplitude of excitatory synaptic events evoked by stimulation of the parallel fibers [42]. GROα also blocked the induction of long-term depression of synaptic transmission (LTD) at the parallel fiber to Purkinje neuron excitatory synapse [42]. LTD is a form of synaptic plasticity that plays an important role in motor learning. In contrast, in similar types of studies in rat and mouse cerebellar slices the chemokine SDF-1α (CXCL12) depressed excitatory synaptic events at parallel fiber to Purkinje neuron synapses, an action that involved a reduction in transmitter release from the presynaptic terminals of the parallel fibers [104]. Cerebellar Purkinje neurons, granule cells and glial cells all express CXCR4, the receptor for SDF-1α (i.e., CXCL12)[71].

In studies of cultured Purkinje neurons from rat cerebellum, acute application of high concentrations of CCL2, thought to simulate pathological conditions, increased intracellular resting Ca^2+^ levels and the intracellular Ca^2+^ signal elicited by exogenous application of an agonist for mGluR1 (metabotropic glutamate receptor 1) but depressed action potential generation [134]. Chronic exposure of cultured Purkinje neurons to high concentrations of IL-6, to simulate pathological conditions, altered intrinsic electrophysiological properties, intracellular Ca^2+^ signaling and Ca^2+^ signals evoked by mGluR1 in the Purkinje neurons [84, 85]. Thus, both CCL2 and IL-6 appeared to target similar functions in the Purkinje neurons.

Effects of chemokines and other neuroimmune factors on cerebellar development and cell positioning has also been investigated using both in vivo and ex vivo approaches and rodent models. These studies demonstrated the critical role neuroimmune factors play in cerebellar cytoarchitecture. Chemokines such as CXCL12 and CXCR4 have chemotactic properties that are important in the control of cell number and position and have been shown to play a key role in normal brain development including cerebellar development [6, 128]. During development chemokines, by binding to and activating their cognate receptor, initiate a process referred to as targeted cell migration, an important mechanism in the prominent morphological and structural changes that occur during fetal and postnatal development of the cerebellum. For example, CXCL12, produced and secreted by cells of the pia mater surrounding the cerebellum, through interactions with its cognate receptor CXCR4 expressed on granule cells, direct tangential migration of granule cell progenitors in the external granule layer. Dysfunction in this pathway may be involved in medulloblastoma (a malignant neoplasm) pathogenesis, considered the most frequent brain tumor of childhood [91]. Involvement of CXCL14 in granule cell migration during development has also been reported. However, in this case CXCL14 is thought to be produced by Purkinje cell dendrites in the molecular cell layer and also the Purkinje cell body. CXCL14 expression occurs only during the period associated with granule neuron migration (e.g., postnatal day 8 to 22) but not in the adult cerebellum [95].

TGFβ1, another neuroimmune factor that contributes to the complex mechanisms involved in cerebellar development, is secreted by granule neurons and astrocytes in addition to the meninges of the pia. TGF-β1 has been shown to regulate expression of specific types of potassium channels in the granule neurons and thereby influence not only electrical activity but also differentiation, growth, survival and maturation of the granule neurons [151]. TGF-β2 is also involved in granule cell development where it serves as a growth and survival factor for granule cell precursors [31].

In vitro studies have shown that IL-6 can also impact granule neuron development and viability. When granule neurons were exposed chronically to elevated levels of IL-6 during their development in culture, IL-6 increased the expression of NMDARs and the membrane depolarization and Ca^2+^ signal produced by NMDAR activation [101]. The NMDA subtype of glutamate receptors plays an important role in granule neuron development, excitatory synaptic transmission, and synaptic plasticity. Glutamate is the primary excitatory neurotransmitter in the brain but also can act as a tropic or toxic factor.

In another study using chronic IL-6 treatment of cultured rat granule during development, IL-6 at low doses (thought to reflect physiological conditions) promoted the growth of cultured granule neurons, but at high doses (thought to reflect pathological conditions) produced cell loss by a process that did not involve apoptosis; IL-6 also increased susceptibility to a toxic insult produced by excessive activation of NMDAR [23]. In other studies, IL-6 was shown to protect against NMDAR-mediated toxicity in developing cultured rat granule neurons, actions that were dependent on the IL-6 concentration and the degree of neuronal damage, effects shown to involved suppression of Ca^2+^ release from the intracellular Ca^2+^ stores [97, 125]. Exogenous application of IL-10 also blocked the toxic effects of excess activation of NMDARs by glutamate in cultured rat cerebellar granule neurons, an action that was associated with IL-10 blockade of glutamate-mediated induction of caspase-3 and NF-kβ DNA binding activity [5].

In studies using an in vitro adenoviral gene delivery approach to induce elevated IL-6 expression in cultured cerebellar granule neurons during development, IL-6 reduced granule cell adhesion and migration and increase expression of proteins associated with excitatory synapses (synaptophysin, a major component of synaptic vesicles and marker for synapses, and the vesicular glutamate transporter, which transports glutamate into synaptic vesicles and is also a marker for excitatory synapses [143]).

Studies of transgenic mice that express altered levels of a specific neuroimmune factor in the brain, such as IL-6, CCL2, interferon-α (INFα), interferon-gamma (INFγ), IL-10 or soluble IL-1 receptor among others, have also been used to investigate the role of neuroimmune factors in brain neuropathology [3, 11–13, 15, 19, 27, 37, 47, 54, 65, 71]. These models have provided information on the in vivo consequences of elevated levels of specific neuroimmune factors on cerebellar structure and function (see also section on astrocytes). For example, altered differentiation and morphogenesis during development and altered cerebellar cytoarchitecture was observed in transgenic mice in which IFNγ was overexpressed selectively by astrocytes, a normal cellular source for this cytokine; in addition, the mice showed ataxia and died at an early age [65]. In another study of IFNγ transgenic mice with targeted astrocyte overexpression, IFNγ induced tumorigenesis including a high incidence of medulloblastoma in the cerebellum, a condition thought to involve immature cerebellar granule neuron precursors [72, 142]. In another transgenic model, IL-6 transgenic mice, over expression of IL-6 by astrocytes resulted in (depending on the level of expression) increased numbers of microglia and astrocytes in the cerebellum (i.e., gliosis), neurologic dysfunction including tremor and ataxia, cerebellar neurodegeneration in older animals, altered protein expression and altered synaptic function [15, 17, 19, 47, 83, 90].

One caveat for studies using transgenic models, which can also apply to other models, is that the neuroimmune factor that was targeted for elevation in the brain of the transgenic mice could induce production of other neuroimmune factors from the same or neighboring cells. This situation makes it difficult to identify the specific neuroimmune factor, cell type mechanisms underlying the observed differences between the transgenic and control mice. The operation of the neuroimmune system typically involves simultaneous production of multiple factors, a complexity that is a challenge for research in this area.

## Cells of the neuroimmune system

In adults, microglia and astrocytes are considered the primary cell types that comprise the neuroimmune system. Two other glial cell types are also present in the brain, oligodendrocytes and NG2 glia (also known as oligodendrocyte precursor cells). These cell types have been shown to produce neuroimmune factors and to contribute to typical actions of the neuroimmune system [149, 150]. However, the information on the neuroimmune actions of these cell types is still limited and they are not discussed in this article.

Microglia and astrocytes are distributed throughout the brain, share many common traits and have overlapping functions. They can display marked brain region dependent phenotypic diversity both across and within brain regions depending on local requirements of the neurons, other cell types or neuronal networks. Bidirectional signaling has been shown to occur between microglia and astrocytes and between these cell types and neurons and to play an important role in establishing the phenotypic characteristics of microglia and astrocytes, and consequently, the functioning of the neuroimmune system [55, 133]. Environmental conditions such as the presence of regulatory/stimulatory factors, pathogens, neurochemicals released by tissue damage, and other factors also play an important role in defining phenotypic characteristics of microglia and astrocytes.

**Fig. 6:**
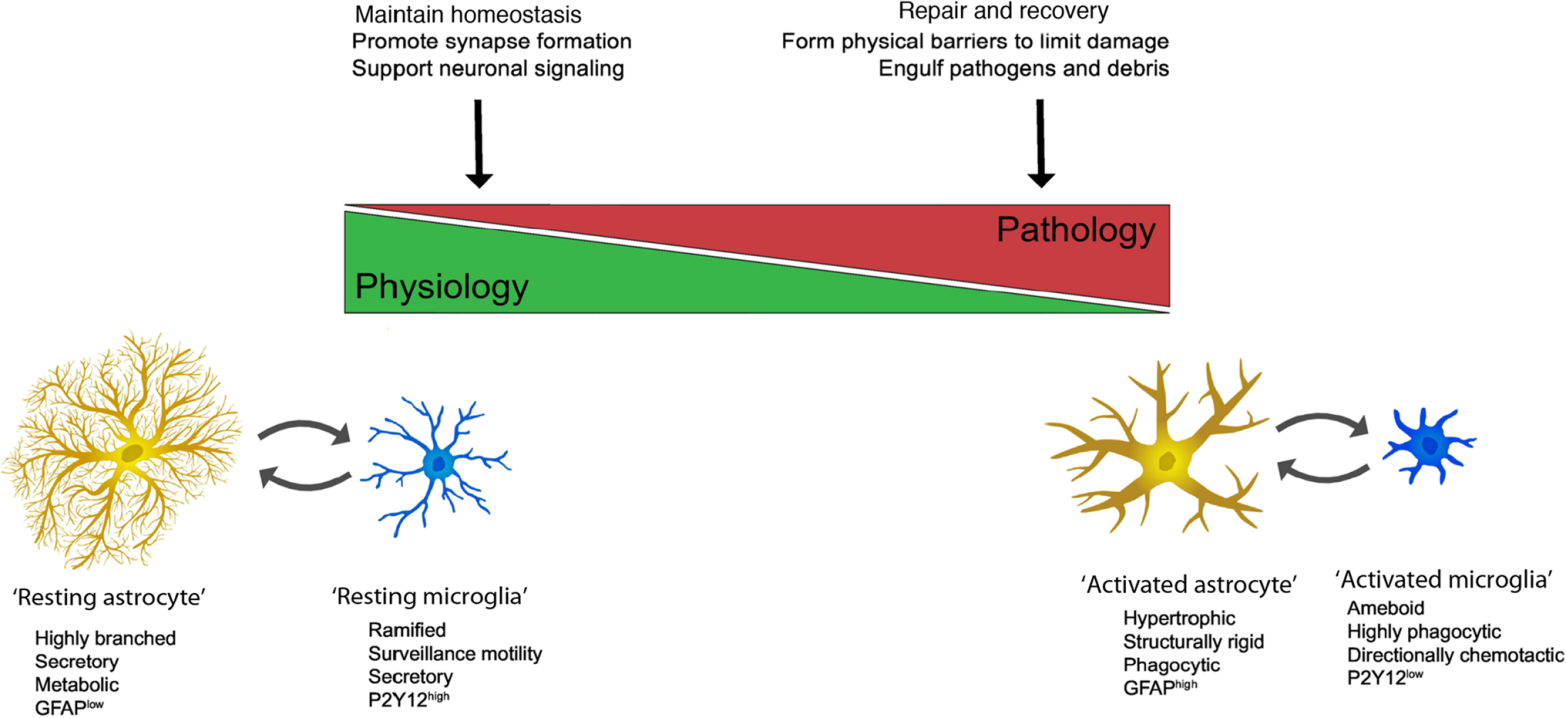
Function of astrocyte-microglia signaling from homeostasis to pathology. Under physiologic conditions (i.e., resting/homeostatic state) astrocytes and microglia support neuronal functions, whereas in pathological conditions (activated/reactive state) they lose some of their supportive functions in favor of optimizing survival. This transition from homeostatic to pathological states is accompanied by a change in morphology and the expression and secretion of molecules associated with the activated/reactive state. Abbreviations: GFAP, glial fibrillary acidic protein, a structural protein primarily expressed by astrocytes in the brain; P2Y12, a purinergic receptor selectively expressed by microglia in the brain. The levels of GFAP and P2Y12 are affected by the state of the astrocyte or microglia, respectively. Arrows signify interactions between these two cell types (reprinted with modification from “Astrocytes and Microglia: In Sickness and in Health” by Vainchtein, I. and Molofsky, A, 2020, Trends Neurosci 43, p. 18 [133])

Classically the phenotypic characteristics of microglia and astrocytes have been considered to fall within one of two states, a ‘resting or homeostatic’ state that is associated with physiological housekeeping functions necessary to maintain the brain in a healthy state, or an ‘activated or reactive’ state that is associated with host defense functions and brain pathology (Fig. 6). While this terminology has been useful, recently studies have questioned its adequacy, as microglia and astrocytes appear to exist in a spectrum of states between resting/homeostatic verses activated/reactive. Consequently, efforts are underway to develop a more comprehensive classification that takes into consideration characteristics of these cells that have been revealed by new techniques (e.g., transcription profiling) [33, 94, 144]. For the purposes of this article, we will use the terms ‘resting or homeostatic’ to refer to the physiological state (i.e., normal healthy state) and ‘activated or reactive’ to refer to pathological states, as new terminology has yet to be firmly established and these terms are used in many relevant articles that may be of interest to readers. Activated/reactive microglia and/or astrocytes in the cerebellum have been described in a number of disease states including Alzheimer’s disease [118], autism [135], Creutzfeldt-Jakob disease [81], Spinocerebellar ataxia type 1 [27] and other ataxias [35, 67], traumatic cerebellar injury [98], and Gerstmann-Sträussler-Scheinker disease [10].

The increasing attention to the phenotypic characteristics (i.e., structure, function, gene expression) of microglia and astrocytes reflects a growing understanding of the role that these cell types play as basic components of the neuroimmune system and the importance of the neuroimmune system to many aspects of normal brain function and development, as well as disease and injury [26, 57, 94, 133, 144]. The availability of new techniques with which to study microglia and astrocytes has been an important factor in the recent progress and has contributed significantly to an understanding of the activities of microglia, astrocytes, their interactions with each other and with neurons, and the biochemical pathways that mediate their functions in normal and pathological conditions [36, 43, 52, 131, 136, 137].

**Fig. 7:**
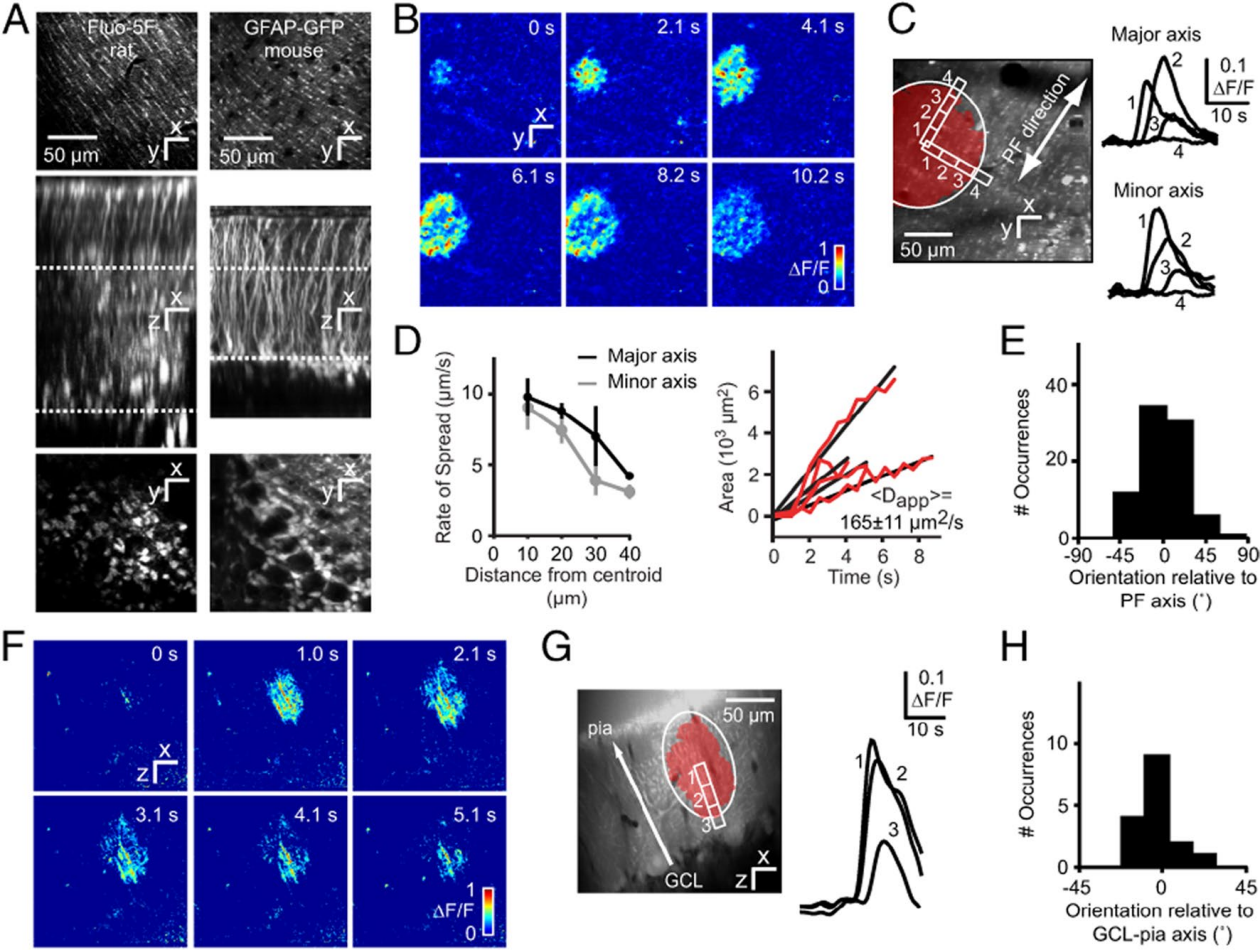
Spontaneous transglial Ca^2+^ waves in the cerebellar cortex of rats or mice in vivo. **A** Staining patterns of the cerebellar cortex bolus-loaded with fluo-5F/AM (rat), a Ca^2+^ sensitive fluorescent dye, or expressing GFP (green fluorescent protein) under control of the glial fibrillary acidic protein (GFAP) promoter (mouse). In the brain, GFAP is primarily expressed in astrocytes. (*Top*) Optical sections acquired in the molecular layer [ML, locations indicated by the upper dotted lines (*Middle*)] show a distinct striate pattern matching lateral protrusions from stem processes of Bergmann glia (BG). (*Middle*) Maximal side projection showing similarity between fluo-5F/AM labeling and GFAP-GFP expression. (*Bottom*) Optical sections taken from the Purkinje cell layer, with BG somata arranged around Purkinje cells. **B** Spontaneous radial wave measured in the ML. **C** Putative stem processes and side branches from BG show calcium increases with a time course typical of glial signals. **D** (*Left*) Wavefront slowing with distance from the initiation site. (*Right*) Linear rate of increase of wave area, with an average apparent diffusion constant *D*app -165_m2/s. Data are shown for 4 waves. **E** Distribution of wave orientation relative to the parallel fiber (PF) axis. **F** Radial wave in ML measured in an *xz* parasagittal plane orthogonal to the surface of the cerebellum. **G** Wave orientation along the axis of BG stem processes. **H** Distribution of wave orientation relative to the pia–Purkinje cell axis.(Reprinted from “Radially expanding transglial calcium waves in the intact cerebellum” by Hoogland, T. et al., 2009, Proc Natl Acad Sci U S A, 106, p. 3497 [53])

For example, studies on the function of microglia and astrocytes have been facilitated by the development of techniques that enable the visualization of live cells in vivo such as microscopic digital imaging combined with newly created non-toxic, fluorescently-tagged chemicals that can identify specific cell types or biological processes in live cells. For example, the development of microscopic Ca^2+^ imaging techniques has enabled visualization of intracellular Ca^2+^ levels in live cells of the brain in vivo or in ex vivo slice and culture preparations. Recent studies using this technique have shown that like neurons, microglia and astrocytes express receptors for a variety of environmental signals such as neurochemicals (e.g., neurotransmitters) and mechanical signals. When the receptors are activated, the cells respond with transient or prolonged changes in intracellular Ca^2+^, a process referred to as Ca^2+^ signaling. In astrocytes Ca^2+^ signals commonly occur as Ca^2+^ waves that travel through astrocytic networks (Fig. 7), while in microglia Ca^2+^ signals typically are localized to processes and occur in a more localized fashion. Ca^2+^ is an important second messenger that regulates numerous biochemical pathways, gene expression and ion channel function. Therefore, changes in the level of intracellular Ca^2+^ can significantly affect the biochemistry, and consequently, the function of microglia and astrocytes.

Much of the microglial Ca^2+^ activity in vivo is thought to occur through metabotropic signaling between neurons and microglia involving ATP (the ligand) and G-protein coupled purinergic receptors. Neurons release ATP during periods of activation, which binds to and activates the metabotropic purinergic receptors on the microglia, which are linked to Ca^2+^ release from intracellular Ca^2+^ stores. In astrocytes Ca^2+^ activity involving glutamate signaling is prominent. Glutamate is released at synaptic terminals during excitatory synaptic transmission and can bind to and activate Ca^2+^ permeable glutamate receptors expressed on astrocytes, which are closely associated with the synapses. Receptor activation results in Ca^2+^ flux into the astrocytes through the glutamate receptor ion channel.

### Microglia

**Fig. 8:**
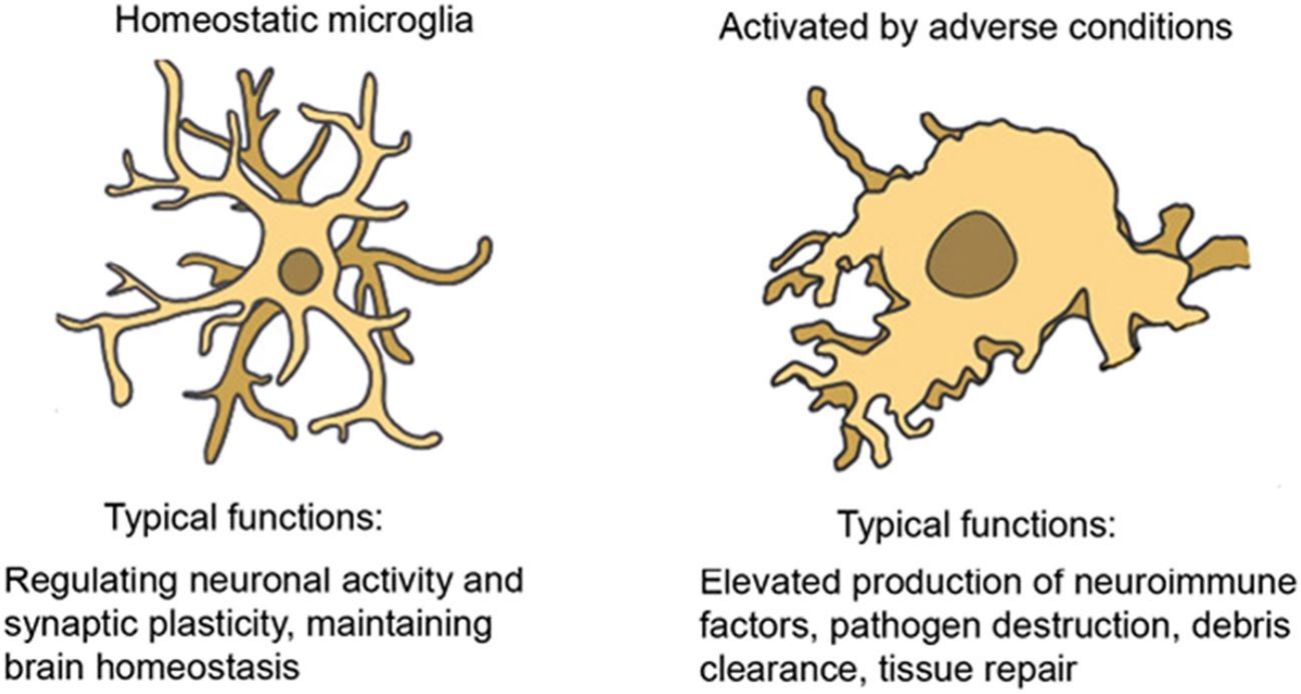
Typical morphological and functional differences between ‘homeostatic’ and ‘activated’ microglia. (Reprinted with modifications from “The semantics of microglia activation: neuroinflammation, homeostasis, and stress” by Woodburn et al., 2021, J Neuroinflammation, p. 258 [144] http://creativecommons.org/licenses /by/4.0/)

Microglia are multifunctional cells that play similar but critical roles across brain regions, with regional variations as a result of neuronal diversity, differences in the chemical and structural microenvironment, hemodynamics and other factors [45]. Under physiological conditions microglia serve as regulators of brain development and aging, participate in the formation and maintenance of neuronal networks, are regulators of synaptic function, perform basic homeostatic functions such as clearance of pathogens, cell debris, and abnormal proteins from the brain, and assist in the repair process. Microglia are highly motile cells and provide continuous surveillance of brain parenchyma and synaptic contacts. Under adverse conditions microglia carry out functions that are intended to combat the effects of the adverse conditions and repair damage. However, if these functions become dysregulated, activated microglia can contribute to the pathology (Fig. 8).

Research involving new technologies (e.g., RNA sequencing, proteomics, epigenetics, and bioinformatics) have shown that brain microglia are embryonically derived from mesodermal progenitor cells in the yolk sac, cells that are also a source of hematopoietic stem cells. During embryogenesis the hematopoietic stem cells in the yolk sac travel to the embryo where they populate the bone marrow. From this population, microglial progenitors evolve, travel to and populate the developing brain where they eventually complete the differentiation process to become mature microglia [70, 121].

A study brains from ~ 33 mammalian species (primarily adult animals) showed that microglia are distributed throughout the brain at similar densities within brain regions and across brain regions. However, they constitute only a relatively small minority of all brain cells, approximately 7% of the non-neuronal brain cells in adult animals [29]. In contrast to the similarity in density of microglial across brain regions, the number of neurons vary widely across brain regions, with the cerebellum having a large number of neurons due to the large granule neuron population. Consequently, the cerebellum has a lower percentage of microglial cells relative to the total cerebellar cell population than for other brain regions. For example, microglial cells comprise approximately 5.8 ± 0.4% of the total number of cells in the cortical region of the brain but only 1.5 ± 0.2% of the total number of cells in the cerebellum. This percentage translates to ∼3 microglial cells per neuron in the cortex of large non-primate brains but only ~ 1 microglial cell per > 100 neurons in the cerebellum of several species [29].

In a healthy brain under normal circumstances, microglia replacement/replication occurs at a low rate and by clonal expansion; although under some circumstances microglia can replicate by replacement from macrophages recruited from the blood [99]. Recent studies using an innovative multicolor fluorescence fate mapping system to chronically monitor microglial dynamics in the brain of mice under homeostatic conditions revealed that the microglial network in the cerebellum, hippocampus and cortex of the adult mouse showed considerable stability and a low rate of microglial self-renewal [110]. Microglia were estimated to completely turn over within 20 months in the cerebellum compared to 15 months in the hippocampus and 41 months in the cortex [110]. However, microglia can rapidly increase proliferation in response to adverse environmental conditions [54].

Recent research has revealed the complexity of microglia and has led to new concepts regarding microglia morphology, physiology, chemistry and function [22, 25, 94]. Microglia exhibit diverse morphological and functional states that can vary with brain region, species, age, sex, environmental factors and context (e.g., health or disease). Recent research has also shown that cerebellar microglia differ from microglia in other brain regions (e.g., cortex) in a variety of characteristics including morphology, motility, gene expression and immune function among other differences [45, 122, 126, 132]. Microglia express a variety of genes and proteins that are relatively unique to and dependent on their state. For example, microglia express receptors that enable them to identify molecular patterns in their environment that are associated with pathogens (PAMPs; e.g., LPS) or tissue damage (DAMPs; e.g., denatured intracellular nuclear or cytosolic proteins), and to phagocytose or endocytose the damaged cells or abnormal proteins and thereby clear their environment [22].

**Fig. 9:**
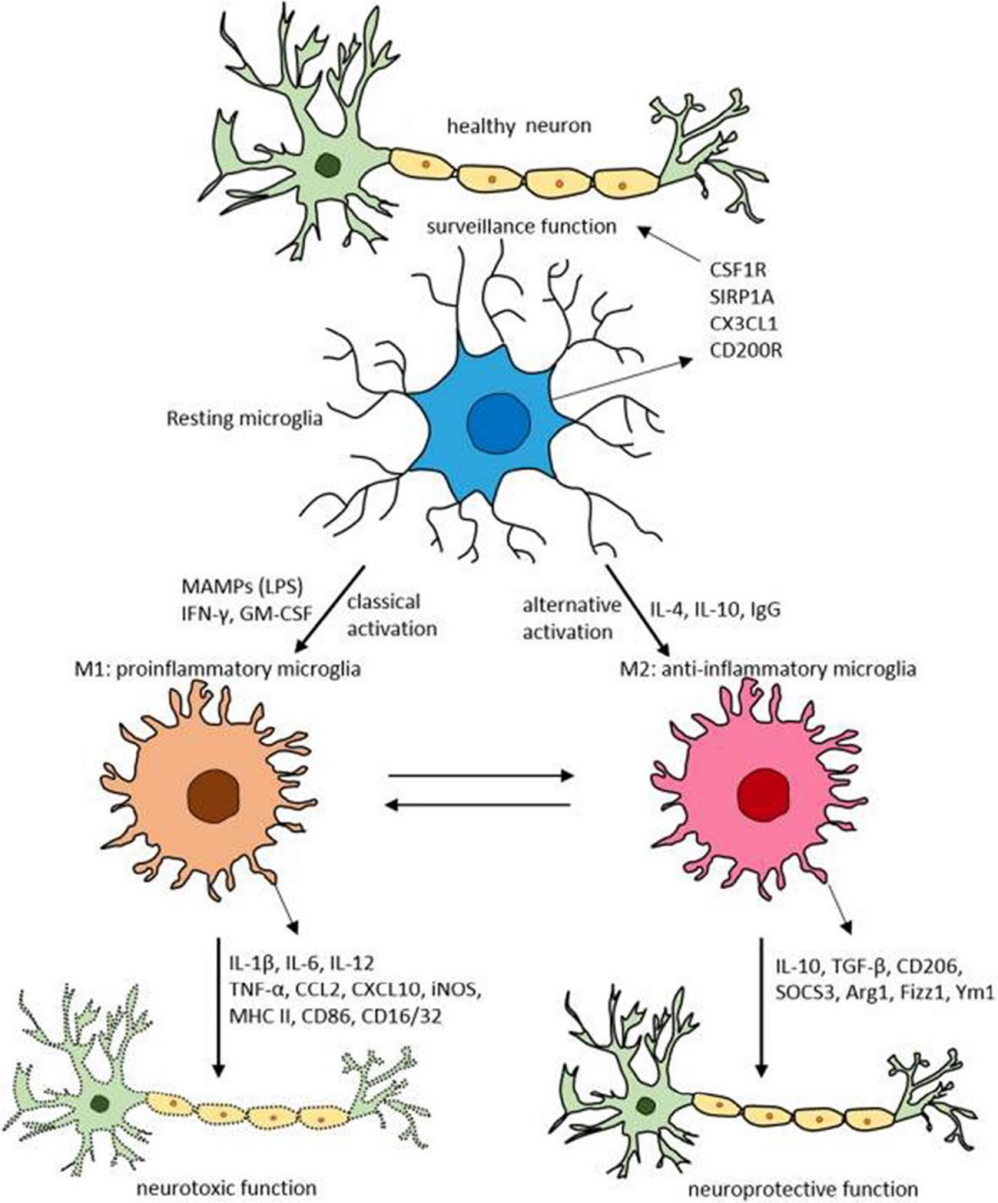
Diagram illustrating microglial activation states M1 and M2, the neuroimmune factors associates with these states and consequences to the target neuron. Abbreviations: CSF1R colony stimulating factor 1 receptor; SIRP1A, signal regulatory protein CD172, chemokine CX3CL1, and CD200R, a type-1 cell membrane glycoprotein of the immunoglobulin supergene family; Arg1, arginase 1; CCL, chemokine (C–C motif) ligand; CD, cluster of differentiation; CSF1R, colony stimulating factor 1 receptor; CXCL, chemokine (C-X-C motif) ligand; Fizz1, found in inflammatory zone; IL, interleukin; GM-CSF, granulocyte–macrophage colony-stimulating factor; IFN-γ, interferon-γ; iNOS, inducible nitric oxide synthase; MAMPs, microbe-associated molecular patterns; MHC-II, major histocompatibility complex II; SIRP1A, signal regulatory protein CD172; SOCS3, suppressor of cytokine signaling-3; TNF-α, tumor necrosis factor-α; Ym1, chitinase-like protein. (Reprinted from “Targeting Microglial Activation States as a Therapeutic Avenue in Parkinson's Disease” by Subramaniam, S. and Federoff, H., 2017, Front Aging Neurosci, 9, p.1 [124])

Several terms have been used to describe microglial states, a subject that has aroused considerable attention and discussion in an effort to achieve consensus as to the most appropriate nomenclature [94]. As noted above, classically microglia have been considered to exist in two states referred to as ‘resting/homeostatic’ or ‘activated/reactive’. More recently the activated/reactive state has been characterized as ‘M1 or M2’, where M1 refers a state associated with a defense response (i.e., an inflammatory response) and the production pro-inflammatory cytokines, and M2 refers to a state associated with adverse or pathological conditions and considered to be an anti-inflammatory and healing state that promotes the release of anti-inflammatory cytokines [22, 124] (Fig. 9).

However, new technological developments such as in vivo imaging of specific brain cell types, has made it evident that the M1/M2 classification for microglia is not sufficiently detailed and that microglia can exist in multiple states that differ with respect to morphological features, the functions performed, and gene and protein expression including the types of neuroimmune factors produced. A discussion of the more complex microglial states is beyond the scope of this article. Thus, as noted above, a two state terminology will be used in this article where “resting or homeostatic” is used for states associated with normal/healthy (e.g., physiological) conditions and ‘activated or reactive’ is used for states associated with conditions that are adverse or pathological.

**Fig. 10:**
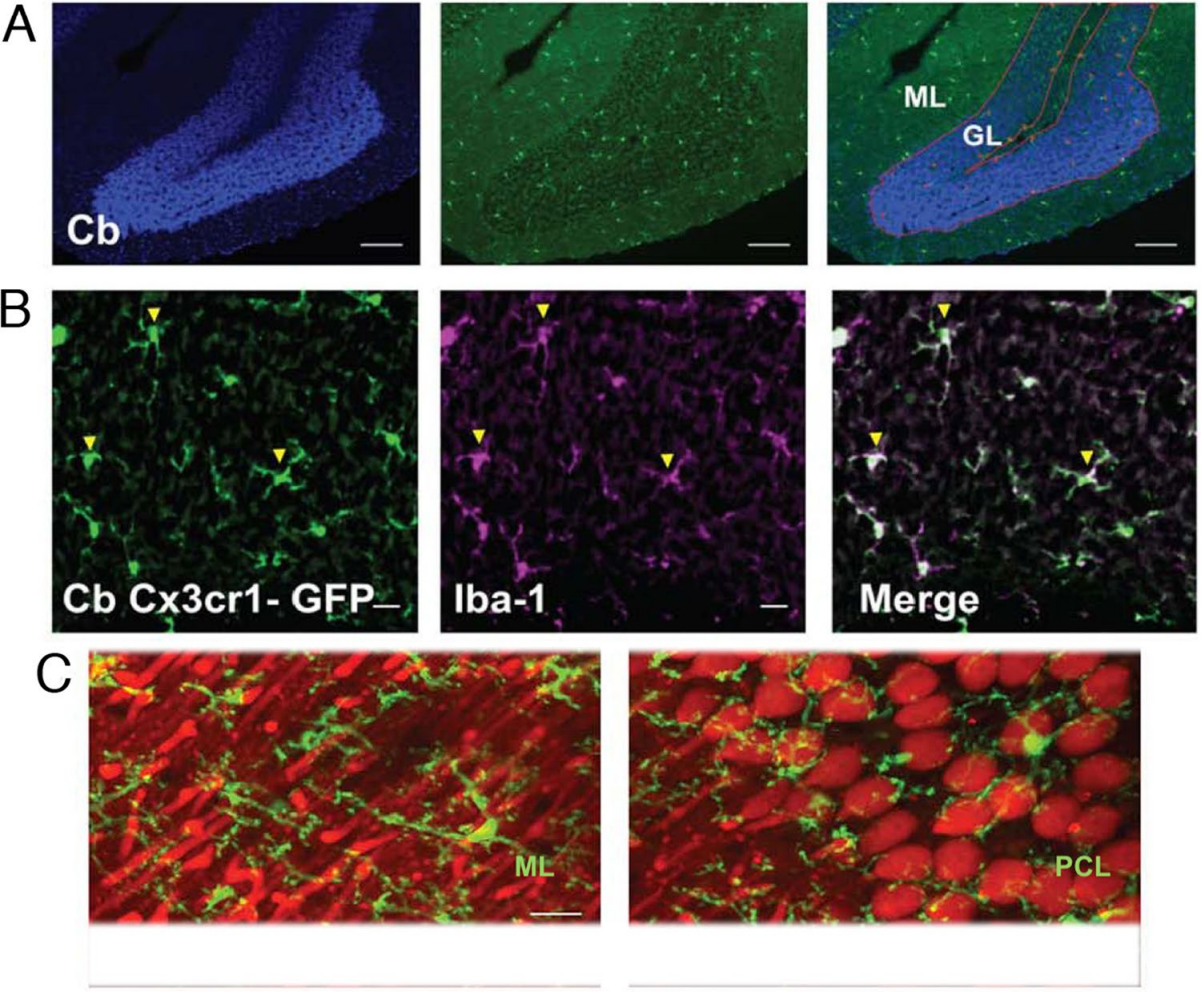
Cerebellar microglia. **A** Epifluorescent images of fixed sections from cerebellum of CX3CR1-GFP mice showing cell bodies (DAPI – blue, left panel) and microglia (GFP – green, middle panel) and merged image (right panel). **B** Confocal images of microglia in fixed sections of cerebellum in CX3CR1-GFP mice showing GFP fluorescence (green, left panel) and microglia immunostained with an antibody to Iba-1, a microglia protein that is commonly used as a marker for microglia (magenta, middle panel), and the merged image (right panel). Examples of co-labeled cells are indicated by yellow arrows. **C** Two-photon laser-scanning microscopic in vivo images showing microglia (green) surveillance of Purkinje neuron (red) dendrites (left panel) and somas (right panel) in cerebellum from mice bred from a cross between the transgenic line Ai9/L7-cre where all Purkinje neurons express tdTomato and the CX3CR1-GFP line. ML = molecular layer; GL = granule neuron layer. Scale bars = 100um (A); 20 μm (B,C). (Reprinted with modification from “Cerebellar microglia are dynamically unique and survey Purkinje neurons in vivo” by Stowell, R. D. et al., 2018, Dev Neurobiol, 8, p.627)

In the homeostatic state, microglial typically have a small somata from which numerous thin and highly ramified processes extended. The processes are very dynamic, extending and contracting as they survey the surrounding cells, synapses and environment. In the activated state, microglia typically transform to a more ameboid morphology associated with phagocytosis of damaged tissue (see Fig. 8). Time-lapse in vivo imaging experiments of adult microglial in the cortex of the brain have shown that that in the homeostatic state, somata of microglia show only minor movement (1 to 2 μm per hour), whereas their processes continuously exhibit cycles of de novo formation and withdrawal (within a time scale of minutes) as they survey the surround, actions that result in morphological changes in the microglia [87]. In a study that compared characteristics of cortical and cerebellar microglia using two-photon imaging in live transgenic mice with fluorescently labeled microglia (GFP label) and neurons (tdTomato label), cerebellar microglia exhibited less ramified microglial arbors and had a greater rate of somatic mobility than microglial in the cortex [122]. Cerebellar microglia were also observed to have diverse but dynamic and intimate interactions with Purkinje neuron dendrites that typically lasted between 5 to 60 min. Interactions between microglia and Purkinje neuron somas were also observed (Fig. 10).

**Fig. 11:**
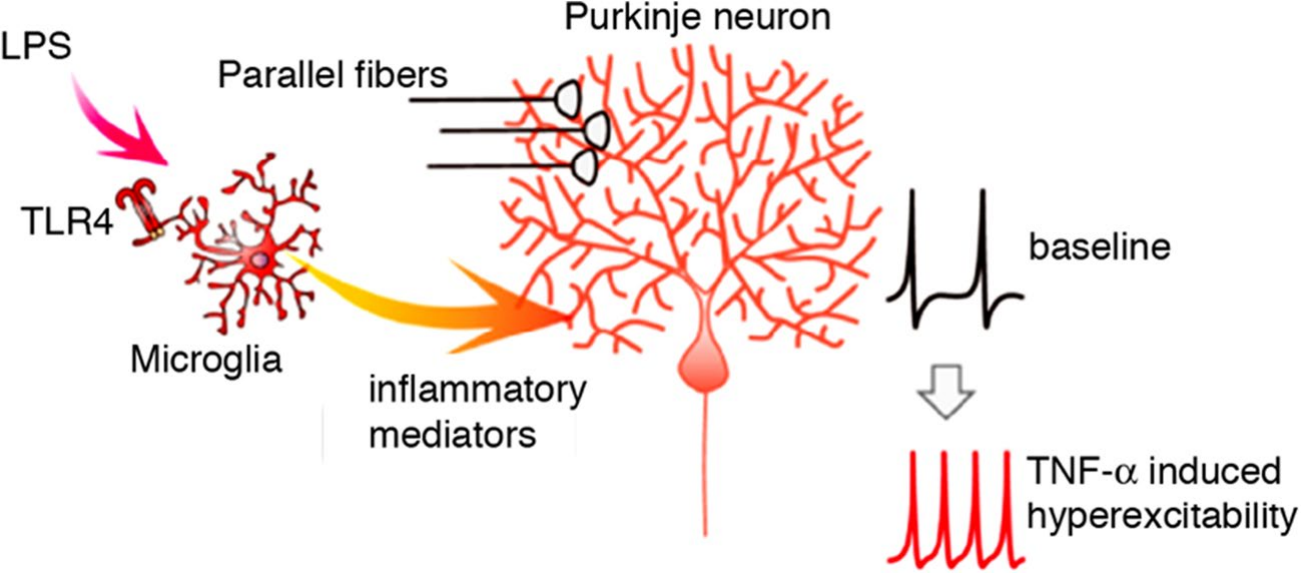
Diagram showing the pathway from LPS activation of microglia through toll-like receptors to changes in Purkinje neuron activity. LPS is used experimentally as a substitute for a pathogen (e.g., virus, bacteria, fungus, etc.). The activated microglia release inflammatory cytokines (e.g., TNF-α, IL-1β, IL-6, etc.) which increased the intrinsic excitability of Purkinje neurons and modulated presynaptic release of the excitatory transmitter glutamate from the parallel fibers and postsynaptic responsiveness of the excitatory synapses on the Purkinje neuron dendrites. (Reprinted with modifications from “Microglia-triggered plasticity of intrinsic excitability modulates psychomotor behaviors in acute cerebellar inflammation” by Yamamoto, M., Kim, M., Imai, H., Itakura, Y., and Ohtsuki, G., 2019, Cell Rep, 28 [146])

Recent studies have also demonstrated that factors released by microglia can alter the electrical activity of Purkinje neurons. For example, in a recent in vitro study of Purkinje neurons in cerebellar slices, microglia activation by application of LPS, an antigen (a PAMP) normally found on the cell surface of most gram-negative bacteria that binds to Toll-like receptor 4 expressed on microglia, increased excitability of Purkinje neurons through a complex mechanism involving TNFα and ATP; parallel in vivo studies showed that the microglia activation resulted in altered behavior (Fig. 11) [146]. In another study, chemogenetic activation of microglia in the cerebellar vermis in vivo resulted increased firing of Purkinje neurons, increased expression of TNF-α, IL-1β and CCL-2 and produced ataxia in mice [145]. Results suggested that the increased excitability of the Purkinje neurons was primarily a result of an action of TNFα on Purkinje neurons [145].

The role of microglia in the cerebellum has also been studied using microglial deficient mice. For example, in one study, selective deletion of cerebellar microglia was achieved by cell-selective genetic manipulation involving an antibody to CX3CR1, a chemokine receptor that is highly expressed in microglia of the cerebellum [82]. The absence of microglia in this study resulted in significantly impaired GABAergic transmission to Purkinje neurons and impaired climbing fiber elimination [82]. In another study, mice that were genetically modified to produce a CSF-1 deficiency in the cerebellum also resulted in depletion of cerebellar microglia. CSF-1 and its receptor CSF1-R play a critical role in proliferation, differentiation, and survival of cerebellar microglia. In the CSF-1 deficient mice, cerebellar structure was altered including loss of Purkinje neurons and altered dendritic structure of surviving Purkinje neurons; in parallel behavioral studies deficits in motor learning and social behavior were observed (i.e., deficit in social memory) [58]. In a study of a mouse model of spinocerebellar ataxia type 1 (SCA1), depletion of microglia from the cerebellum during an early stage of the disease using an inhibitor of CSF-R1 resulted in reduced expression of TNF-α, an effect that was associated with improved motor function [102].

### Astrocytes

Astrocytes are one of the most abundant cell types in the brain, although the exact numbers, which vary with brain region, and neuron to astrocyte ratio is still under investigation [141]. It has been reported that astrocytes make up between 17 and 61% of the cells in the human brain, depending on the brain region [41]. Astroglial density has been estimated to be ~ 600 cells/mm^−2^ in 30 μm sections of the cerebellar folia of adult mice compared to ~ 80 cells/mm^−2^ in 30 μm sections of the cerebral cortex. The proliferation rate, as assessed by mitotic index (BrdU-positive astroglia as a percentage of astroglia labeled by a GFAP antibody), was 2–3% of the total astrocytes in the cerebellum compared to ~ 9% of the total astrocytes in the cerebral cortex and ~ 11% of the total number of astrocytes overall for the entire brain [32].

Classically, astrocytes have been considered primarily support cells for brain cytoarchitecture and caregivers of brain health (e.g., by providing metabolic support, maintaining ion balance, clearing transmitters from the synaptic environment, regulating the blood brain barrier). However, it is now known that astrocytes perform a variety of additional activities both in the developing and mature brain and that these activities are essential for the establishment and maintenance of functional cognitive and control systems that oversee behavior [3, 18, 21, 93, 137]. For example, astrocytes regulate neuronal excitability and synaptic function, control extracellular potassium and glutamate concentrations, and perform neuroimmune functions. During development astrocytes regulate neurogenesis, establish and maintain brain architecture, direct cell migration and differentiation, and regulate synaptogenesis and synaptic pruning. Cerebellar structure is severely disrupted if astrocytes are ablated early in development [9]. Significant alterations in cerebellar structure during development or in the adult results in ataxia [18].

**Fig. 12:**
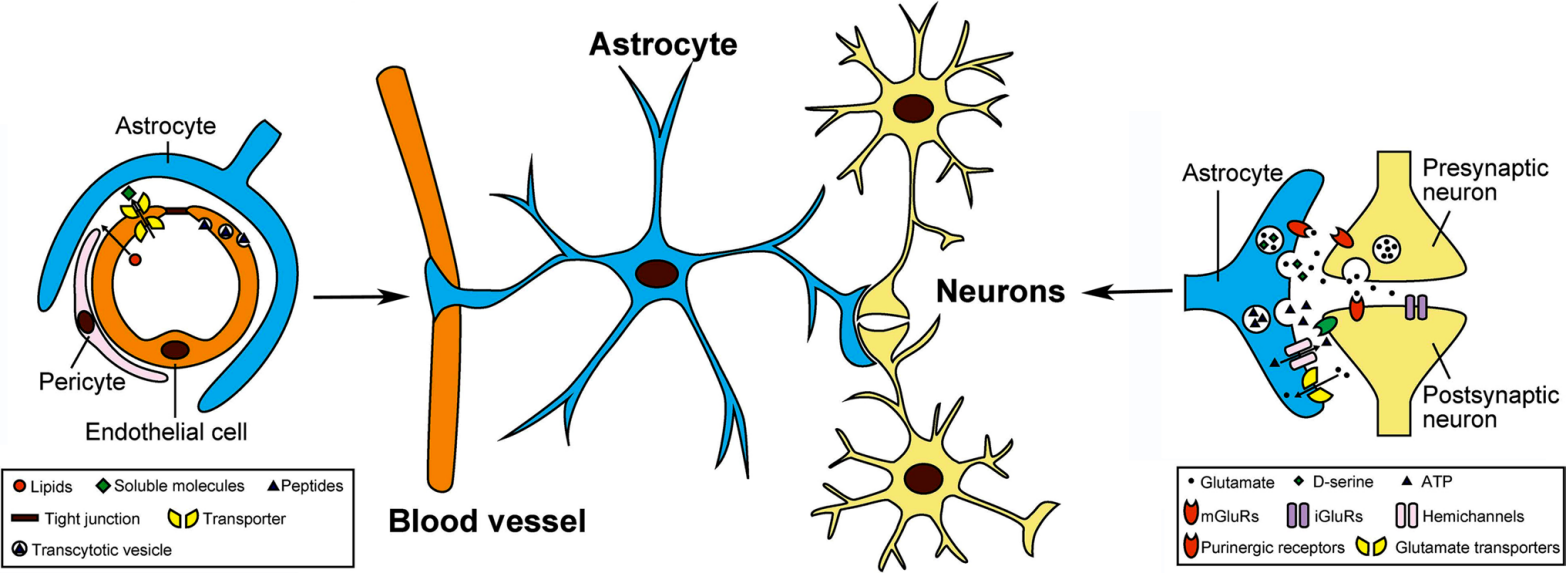
Diagrams showing close morphological and functional associations between astrocytes and synapses and blood vessels. Astrocytes are an important component of the blood brain barrier that regulates infiltration of blood born chemicals and cells into the brain. Astrocytes are also part of a tripartite synapse where complex signaling interactions mediated by neurotransmitters (e.g., glutamate) and gliotransmitters (e.g., glutamate, D-serine, and ATP) occurs. Neurotransmitters are released from presynaptic terminals of neurons and act at receptors on post synapses of neurons and on astrocytes. Astrocytes release gliotransmitters such as glutamate that act at receptors on presynaptic and postsynaptic terminals of neurons. Integration of these signaling actions modulate synaptic transmission and plasticity. Astrocytes also regulate extracellular glutamate levels through the actions of glutamate transporters. (Reprinted with modification from “Gliotransmitter Release from Astrocytes: Functional, Developmental, and Pathological Implications in the Brain” by Harada, K. et al., 2015, Front Neurosci, 9, p. 499 [33])

**Fig. 13:**
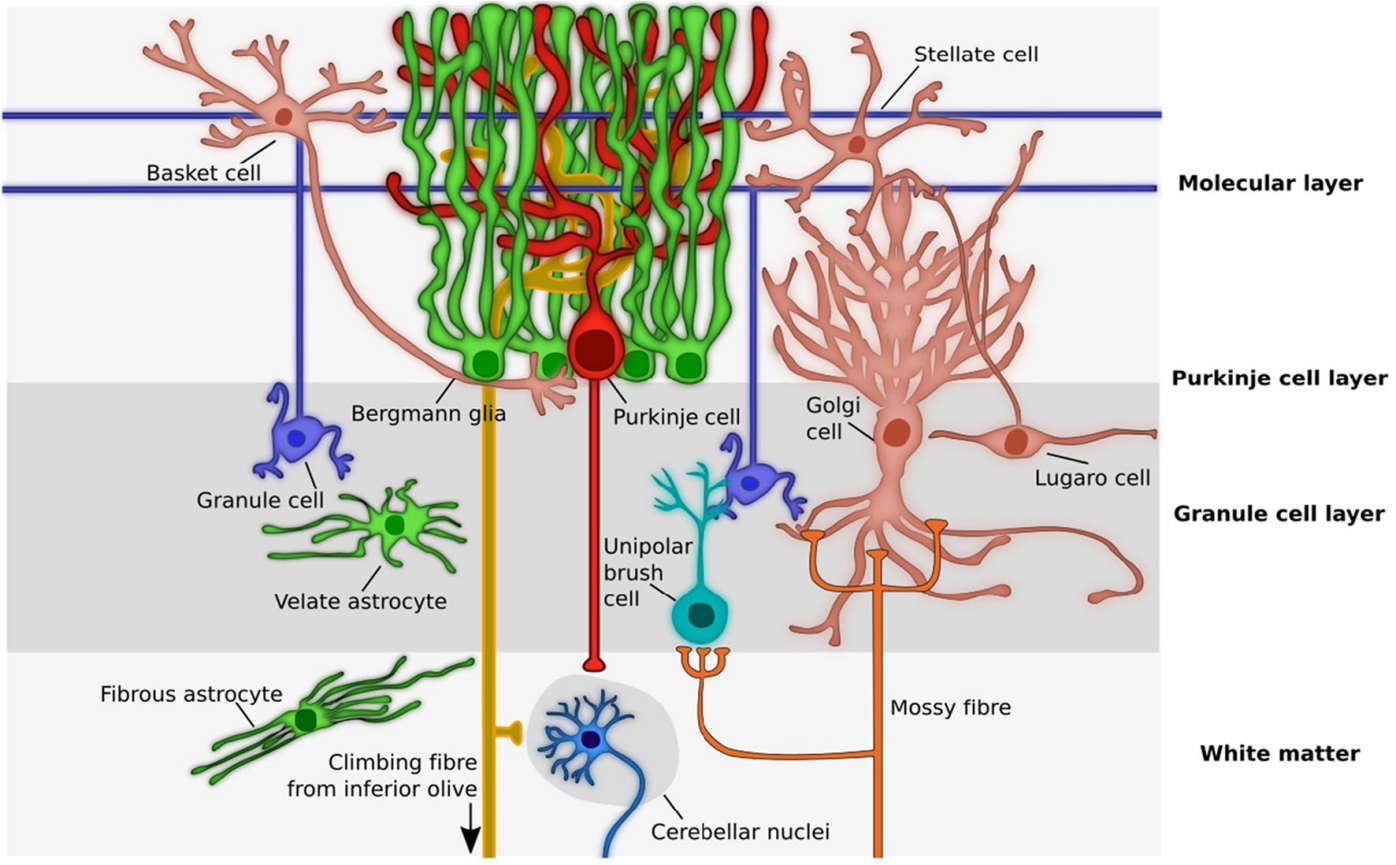
Diagram showing cerebellar neuron and astrocyte cell types and their placement in cerebellar architecture. Neurons = granule neuron, Purkinje neuron, Unipolar brush cell, Lugaro cell, Golgi cell, Basket cell, Stellate cell, neuron of cerebellar nuclei. Astrocytes = Fibrous astrocyte, Velate astrocyte, Bergman glia. (Reprinted from “Cerebellar Astrocytes: Much More Than Passive Bystanders In Ataxia Pathophysiology” by Cerrato, V., 2020, J Clin Med, 9, p. 757 [18])

Astrocytes are closely associated with cells in their environment including other astrocytes, microglia, neurons and cells that form blood vessels (e.g., endothelial cells), where they are an important component of the blood–brain barrier (Fig. 12). A single astrocyte can make contact with multiple neurons and blood vessels through their numerous processes. Astrocytes contact soma, dendrites, and synaptic terminals of neurons. Bidirectional communication occurs between synaptic terminals and astrocytic process and plays an important role in the regulation of synaptic transmission and plasticity both in normal and pathological conditions (Fig. 13). Astrocytes form widespread signaling networks with other astrocytes through connections at gap junctions located on astrocyte processes.

Developmentally astrocytes are derived from progenitor cells in the ventricular zone of the brain and from this site migrate to various brain regions, including the cerebellum, where they complete morphogenesis and final placement. The majority of cerebellar astrocytes are generated between the embryonic and late postnatal period. Astrocytes do not appear to replicate in the normal adult mouse cerebellum [123] except under pathological conditions but can be replenished from progenitor cells produced in the cerebellum. Cerebellar astrocytes are commonly classified into four main groups based on morphology and locations in cerebellar architecture: fibrous astrocytes are located in the white matter, stellate multipolar astrocytes or protoplasmic astrocytes are located in the granular cell layer, and Bergmann’s glia are located between the Purkinje cell layer and the molecular layer. Bregman glia are unique to the cerebellum, are derived from radial glia and are considered to be specialized type of astrocytes (Fig. 13).

**Fig. 14:**
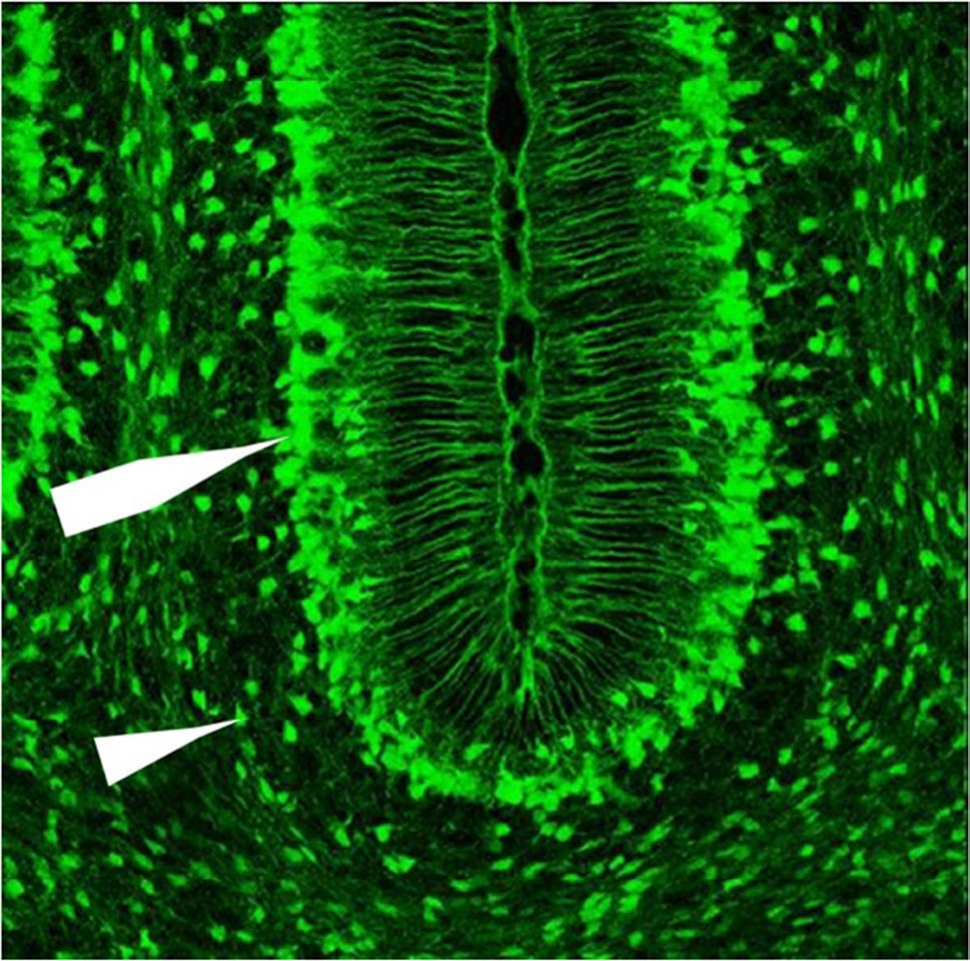
Confocal image of EGFP (enhanced green fluorescent protein) expression in the cerebellum of a postnatal day 7 Slc1a3 (glial high-affinity glutamate transporter; EAAT1) transgenic mouse. EGFP expression is observe in Bergmann (large arrow) and scattered astrocytes (arrowhead).(Reprinted with minor modification from The Gene Expression Nervous System Atlas (GENSAT) Project, NINDS Contracts N01NS02331 & HHSN271200723701C to The Rockefeller University (New York, NY)

Bergman glia have been the most studied of the cerebellar astrocytes, particularly with respect to neuron to glial signaling, which is important during development for cell migration and synaptogenesis and in the adult for maintaining the proper function of excitatory synaptic transmission by controlling the levels of glutamate around synapses (i.e., glutamate homeostasis). Less is known about the function of other types of cerebellar astrocytes, they appear to play typical astrocyte roles (e.g., regulation of tissue homeostasis, functioning of cerebellar circuits) but may also have unique functions [9, 18, 52, 63]. Glutamate is the main excitatory transmitter in the brain including at the parallel fiber and climbing fiber synapses to Purkinje neurons. During intense excitatory synaptic transmission extracellular glutamate levels can increase at the synapse and if not regulated can become toxic resulting in damage and cell death. Bergman glia are closely associated with Purkinje neurons (see Fig. 13) and under normal conditions protect Purkinje neurons from glutamate toxicity through the action of glutamate transporters EAAT1 (GLAST) and EAAT2 (GLT-1). The highest number of EAAT1 in the cerebellum are expressed by Bergman glia [66](Fig. 14).

Glutamate transporters transport glutamate from the extracellular environment into astrocytes for conversion to glutamine. Glutamine is a precursor for the excitatory neurotransmitter glutamate and the inhibitory neurotransmitter GABA and is transported out of the astrocytes and taken up by neurons for this purpose. Bergman glia can also store glutamate and release it in a Ca^2+^ dependent manner. The released glutamate can then act at glutamate receptors on Purkinje neurons and influence synaptic response to excitatory synaptic input from the parallel or climbing fibers to the Purkinje neurons.

**Fig. 15:**
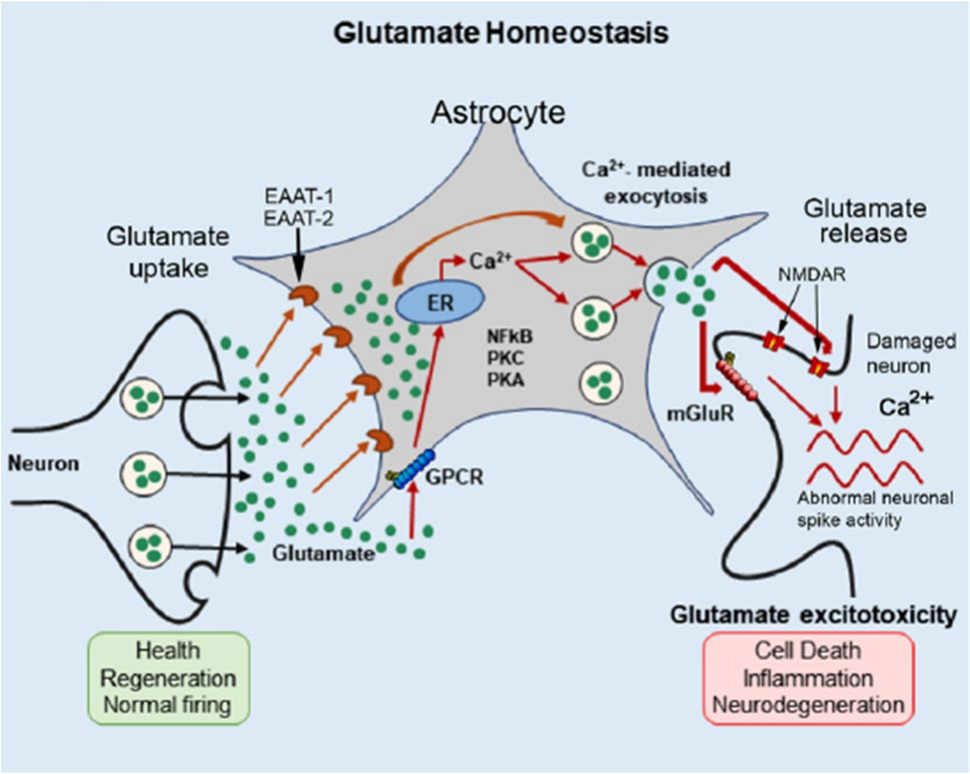
Astrocytes and glutamate homeostasis. Glutamate is released from presynaptic terminals of excitatory neurons and acts at glutamate receptors on the postsynaptic neuron (not shown). Some of the released glutamate spillovers into the extracellular fluid where it is taken up by astrocytes, which converted it into glutamine by glutamine synthetase or stored in vesicles. Glutamine is released to the extracellular space and taken up by neurons for use in the production of glutamate or GABA. Under conditions of intense synaptic activity or when injury to neurons occurs, excess release of glutamate can increase the glutamate concentration in the extracellular fluid resulting in excess activation of glutamate receptors (mGluR1, NMDAR) on neurons. The glutamate receptors are linked to increases in intracellular Ca^2+^, either through influx from the extracellular fluid (i.e., NMDAR) or Ca^2+^ release from intracellular stores (mGluR1) resulting in Ca.^2+^ induced neuronal toxicity. (Reprinted with modification from “Astrocytes Maintain Glutamate Homeostasis in the CNS by Controlling the Balance between Glutamate Uptake and Release” by Mahmoud, S. et al.,2019, Cells 8, graphical abstract)

During excitatory (glutamatergic) synaptic transmission to Purkinje neurons there is spillover of glutamate from the synapse (Fig. 15). In addition, extrasynaptic release from presynaptic terminals is thought to occur. Glutamate from both of these sources can activate glutamate receptors on Bergman glia including Ca^2+^ permeable AMPARs. Activation AMPARs results in Ca^2+^ influx through the AMPAR channel and increases in intracellular Ca^2+^ levels. In Ca^2+^ imaging experiments of live Bergman glia in vivo, stimulation of climbing or parallel fibers by motor activity resulted in Ca^2+^ transients in the Bergman glia, a response that also occurs when exogenous glutamate is acutely applied to cerebellar slices [53, 78], Spontaneous Ca^2+^ waves produced by ATP and acting through purinergic receptors have been also observed in Bergmann glial cells in vivo [53, 78]

Excess excitatory synaptic activity resulting in elevated levels of extracellular glutamate can induce neuronal toxicity, which occurs primarily through increased intracellular Ca^2+^ levels (Fig. 15). Glutamate toxicity can be impacted by neuroimmune factors produced in response to the adverse conditions associated with glutamate toxicity. For example, studies in acutely isolated cerebellar slices from postnatal rats showed that TNF-α increased glutamate-induced excitotoxicity in Purkinje neurons resulting in increased cell death, an action that involved an increase in intracellular Ca^2+^ in the Purkinje neurons mediated by Ca^2+^ permeable membrane receptors [8].

Like microglia, astrocytes can exist in a ‘resting/homeostatic’ or ‘activated/reactive’ state. Formation of the activated state is associated with alterations in gene expression, morphology, proliferation, and function. Activated astrocytes are often referred to as ‘reactive astrocytes’ and can exhibit different phenotypes, for example A1 or A2, with A1 astrocytes exhibiting pro-inflammatory, neurotoxic properties and A2 astrocytes exhibiting neuroprotective properties [26, 33]. As for the M1/M2 classification of microglial activation, recent studies of astrocytes in various neurodegenerative diseases indicate that the A1/A2 classification does not fully reflect the complexity of the astrocyte states. Therefore, further work is necessary in this area. Activation of astrocytes is typically associated with increased production of GFAP (glial fibrillary acidic protein) a key constituent of the intermediate filament of the astrocyte cytoskeleton. Immunostaining with antibodies to GFAP is commonly used as to a marker for astrocytes, with increased expression interpreted as evidence of ‘astrogliosis’ and indicative of adverse conditions and astrocyte activation. Astrogliosis is often taken to mean an increase in astrocyte cell number but the increase in GFAP could also reflect an increase in astrocyte size.

**Fig. 16:**
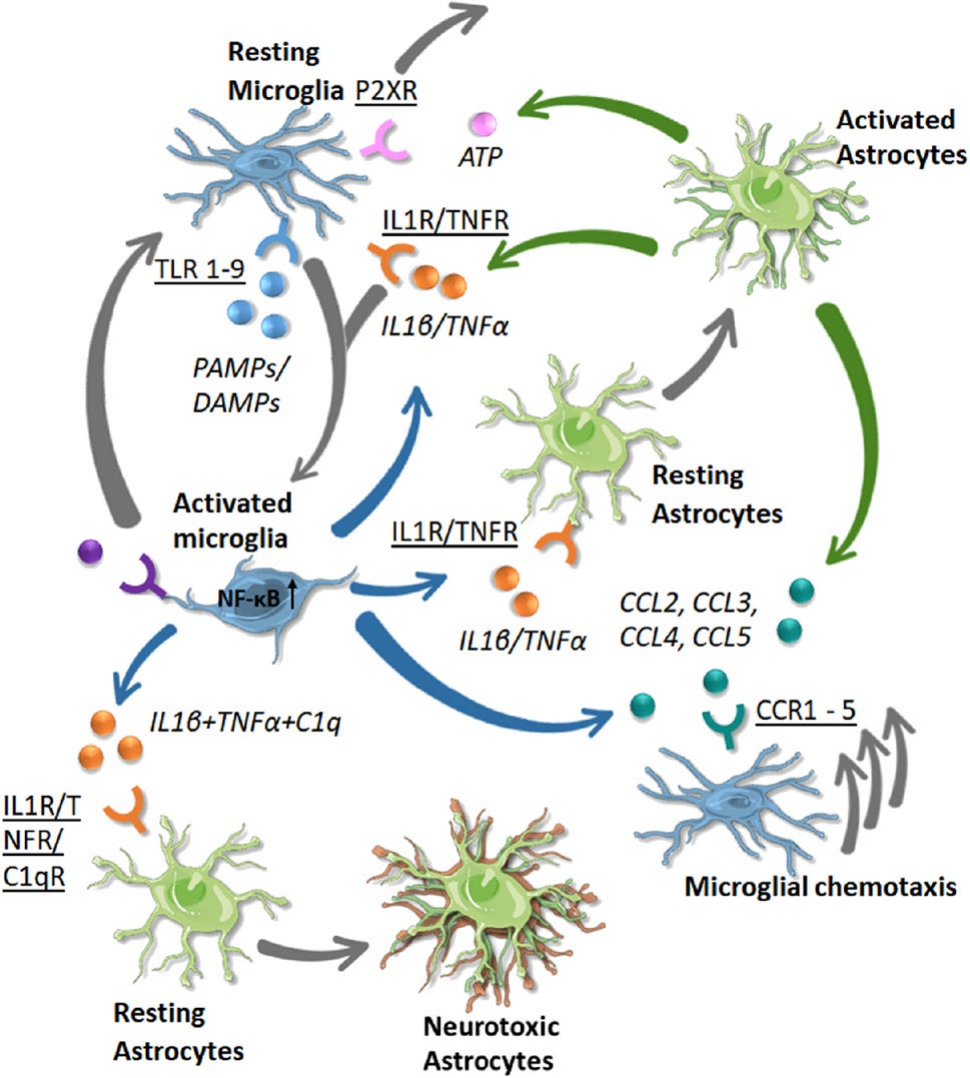
Interactions between microglia and astrocyte during pathological conditions. Receptors (underlined) and the signaling molecules (in italics) are shown with the color of the arrow reflecting the cell source (green = astrocytes, blue = microglia). The resulting phenotypic changes or movements are shown by gray arrows (single or triple, respectively). Activation of microglia is initiated by PAMPs/DAMPs acting at TLR receptors (both blue) on the microglia. As the microglia become activated (gray arrow) they release IL1b and TNFa, which bind IL1R and TNFR to activate astrocytes and other microglial. This signaling with the addition of C1q (a component of the pathway involved in complement activation) on astrocytes leads to a neurotoxic phenotype, with a loss of neuroprotective functions. Activated astrocytes release IL1b and TNFa, which propagates the neuroinflammatory response. Activated astrocytes also release ATP which binds to P2XRs (purinergic receptors) on microglia. Activated astrocytes and microglia may also release chemokines (CCL2–CCL5) which bind to receptors on microglia (CCR1–CCR5) and stimulate their chemotaxis toward the site of injury/pathogen. Inflammation is in part resolved by CD200 binding to CD200R on activated microglia, which suppresses the immune function of microglia steering them toward a homeostatic phenotype. (Reprinted with modification from “Microglia and Astrocyte Function and Communication: What Do We Know in Humans?” by Garland, E. F. et al., 2022, Front Neurosci,16, 1 [41])

Adverse conditions typically produce activation of both astrocytes and microglia with interactions/communication between these two cell types mediated by neuroimmune factors (Fig. 16).

**Fig. 17:**
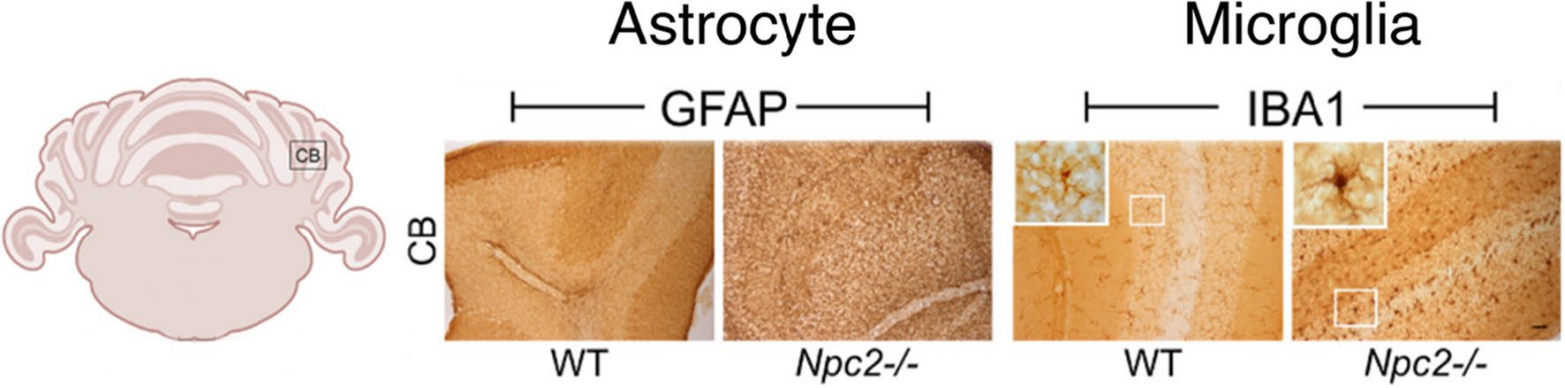
Activated astrocytes and microglia in cerebellum of a mouse model for Niemann-Pick type C disease (Npc2) compared to age matched wildtype (WT) controls. Astrocytes and microglia are immunostained for proteins that are considered specific cell markers, GFAP and Iba-1 (brown product for both), respectively. Insets of immunostained microglia show cellular morphology. Scale bar = 20 μm (50 μm for inset). (Reprinted with modification from “The Npc2.^(Gt(LST105)BygNya)^ mouse signifies pathological changes comparable to human Niemann-Pick type C2 disease” by Rasmussen, C. L. M. et.al., 2023, Mol Cell Neurosci, 126, 1[105])

For example, activation of cerebellar astrocytes and microglia appear to be involved in several forms of ataxia (e.g., [18]). Studies in a mouse model of spinocerebellar ataxia type 1 (SCA1) showed that both astrocytes and microglia were activated at an early stage of the disease, an effect that occurred before neuronal death and could have involved signals from damaged Purkinje neurons [27]. In addition, increased levels of the pro-inflammatory cytokines TNFα, MCP-1 occurred at the early stage of the disease in the SCA1 mice, with the additional increase in levels of IL-6 as the disease progressed [27]. These results suggested that as pathological conditions develop in the SCA1 mice, microglia are activated first and subsequently trigger astrocyte activation through release of ATP and inflammatory mediators such as proinflammatory cytokines (e.g., IL-1, TNFα) or other inflammatory mediators (e.g., prostaglandin E2) [105](Fig. 17).

Detrimental effects of astrocyte activation and elevated levels of astrocyte produced neuroimmune factors has also been demonstrated by studies of transgenic mice that were genetically modified to constitutively produce elevated expression by astrocytes of a specific cytokine, such as IL-6, IL-3, IL-12, INF, or TNF-α. The enhanced astrocyte production of the neuroimmune factors resulted in chronic brain inflammation involving astrocytes and microglia and neuropathology that was unique to the over-expressed cytokine [4, 12, 14, 48, 100]. For example, in the IL-6 transgenic mice the highest level of IL-6 mRNA expression in the brain was in found in the Bergman glia of the cerebellum; low levels of TNFα mRNA and IL-1α/β mRNA were also observed in the cerebellum [19]. These mice were characterized by enhanced cerebellar expression of GFAP, indicative of astrogliosis, Iba1, indicative of microgliosis, and TNFα. In these mice, it is likely that the microgliosis was triggered by the enhanced levels of astrocyte produced IL-6 [48]. The mice also showed altered dendritic structure of Purkinje neurons, reduced number of Purkinje neurons and granule neurons, altered cerebellar synaptic function and synaptic protein expression, impaired motor performance and ataxia that progressed with aging, and disruption of the blood brain barrier [13, 47, 48, 83]. The severity of these characteristics depended on the level of IL-6 expression and the age of the mouse, becoming more severe with higher levels of IL-6 and age. Excessive exposure to alcohol and other drugs is also associated with astrocyte and microglial activation in the cerebellum and ataxia (e.g., [51, 130, 148]).

## Concluding remarks

The neuroimmune system of the brain is a dynamic and complex system that is present in all brain regions and plays an important role in homeostatic processes essential for normal brain function and in repair and recovery processes that restores brain structure and function after damage, disease, and other pathological conditions. The primary cell types that form the neuroimmune system are the astrocytes and microglia of the brain. These cell types have both distinct and unique phenotypes and functions and co-operate in the performance of neuroimmune actions in healthy and pathological states. Microglia and astrocytes achieve their functions primarily through production and secretion of a family of signaling factors commonly used by the peripheral immune system called cytokines, which are referred to as neuroimmune factors when produced by brain cells. Dysregulation of astrocytes or microglia cytokine production during adverse conditions can induce pathology such as occurs in neurodegenerative diseases. Dysregulation of cerebellar astrocytes or microglia cytokine production can lead to pathology and impaired motor function.

Advancing an understanding of the functioning of the neuroimmune system is currently at the forefront of interest in many areas of brain study and will likely continue for a long time into the future due to the expansive role this system plays in brain biology that has been revealed by recent research. Studies of the neuroimmune system of the cerebellum has lagged that of other brain regions. However, recent research has demonstrated the critical areas that the neuroimmune system plays in cerebellar development, function and pathology. Therefore, studies of the cerebellar neuroimmune system is a topic of study that is ripe for scientists interested in uncovering the biological secrets of this new and exciting field where much is yet to be discovered.

## Data Availability

Not applicable.

## References

[1] Agusti A, Hernández-Rabaza V, Balzano T, Taoro-Gonzalez L, Ibañez-Grau A, Cabrera-Pastor A, Fustero S, Llansola M, Montoliu C, Felipo V. Sildenafil reduces neuroinflammation in cerebellum, restores GABAergic tone, and improves motor in-coordination in rats with hepatic encephalopathy. CNS Neurosci Ther. 2017;23:386–94. 28296282 10.1111/cns.12688 PMC6492705

[2] Andoh T, Kishi H, Motoki K, Nakanishi K, Kuraishi Y, Muraguchi A. Protective effect of IL-18 on kainate- and IL-1 beta-induced cerebellar ataxia in mice. J Immunol. 2008;180:2322–8. 18250441 10.4049/jimmunol.180.4.2322

[3] Araujo APB, Carpi-Santos R, Gomes FCA. The Role of Astrocytes in the Development of the Cerebellum. Cerebellum. 2019;18:1017–35. 31218566 10.1007/s12311-019-01046-0

[4] Asgarov R, Sen MK, Mikhael M, Karl T, Gyengesi E, Mahns DA, Malladi CS, Münch GW. Characterisation of the mouse cerebellar proteome in the GFAP-IL6 model of chronic neuroinflammation. Cerebellum. 2022;21:404–24. 34324160 10.1007/s12311-021-01303-1

[5] Bachis A, Colangelo AM, Vicini S, Doe PP, De Bernardi MA, Brooker G, Mocchetti I. Interleukin-10 prevents glutamate-mediated cerebellar granule cell death by blocking caspase-3-like activity, The Journal of neuroscience : the official journal of the Society for. Neuroscience. 2001;21:3104–12. 11312295 10.1523/JNEUROSCI.21-09-03104.2001 PMC6762554

[6] Bajetto A, Bonavia R, Barbero S, Florio T, Schettini G. Chemokines and their receptors in the central nervous system. Front Neuroendocrinol. 2001;22:147–84. 11456467 10.1006/frne.2001.0214

[7] Banjara M, Ghosh C. Sterile Neuroinflammation and Strategies for Therapeutic Intervention. Int J Inflam. 2017;2017:8385961. 28127491 10.1155/2017/8385961 PMC5239986

[8] Bliss RM, Finckbone VL, Trice J, Strahlendorf H, Strahlendorf J. Tumor necrosis factor-α (TNF-α) augments AMPA-induced Purkinje neuron toxicity. Brain Res. 2011;1386:1–14. 21276434 10.1016/j.brainres.2011.01.059

[9] Buffo A, Rossi F. Origin, lineage and function of cerebellar glia. Prog Neurobiol. 2013;109:42–63. 23981535 10.1016/j.pneurobio.2013.08.001

[10] Bugiani O, Giaccone G, Piccardo P, Morbin M, Tagliavini F, Ghetti B. Neuropathology of gerstmann-sträussler-scheinker disease. Microsc Res Tech. 2000;50:10–5. 10871543 10.1002/1097-0029(20000701)50:1<10::AID-JEMT3>3.0.CO;2-6

[11] Cabrera-Pastor A, Llansola M, Montoliu C, Malaguarnera M, Balzano T, Taoro-Gonzalez L, García-García R, Mangas-Losada A, Izquierdo-Altarejos P, Arenas YM, Leone P, Felipo V. Peripheral inflammation induces neuroinflammation that alters neurotransmission and cognitive and motor function in hepatic encephalopathy: underlying mechanisms and therapeutic implications. Acta Physiol (Oxf). 2019;226:e13270. 30830722 10.1111/apha.13270

[12] Campbell IL. Transgenic mice and cytokine actions in the brain: bridging the gap between structural and functional neuropathology. Brain Res Brain Res Rev. 1998;26:327–36. 9651549 10.1016/s0165-0173(97)00038-6

[13] Campbell IL, Abraham CR, Masliah E, Kemper P, Inglis JD, Oldstone MB, Mucke L. Neurologic disease induced in transgenic mice by cerebral overexpression of interleukin 6. Proc Natl Acad Sci USA. 1993;90:10061–5. 7694279 10.1073/pnas.90.21.10061 PMC47713

[14] Campbell IL, Chiang CS. Cytokine involvement in central nervous system disease. Implications Transgenic Mice, Ann New York Acad Sci. 1995;771:301–12. 8597408 10.1111/j.1749-6632.1995.tb44690.x

[15] Campbell IL, Erta M, Lim SL, Frausto R, May U, Rose-John S, Scheller J, Hidalgo J. Trans-signaling is a dominant mechanism for the pathogenic actions of interleukin-6 in the brain. J Neurosci: Off J Soc Neurosci. 2014;34:2503–13. 10.1523/JNEUROSCI.2830-13.2014 PMC6802757 24523541

[16] Campbell IL, Krucker T, Steffensen S, Akwa Y, Powell HC, Lane T, Carr DJ, Gold LH, Henriksen SJ, Siggins GR. Structural and functional neuropathology in transgenic mice with CNS expression of IFN-alpha. Brain Res. 1999;835:46–61. 10448195 10.1016/s0006-8993(99)01328-1

[17] Carrasco J, Hernandez J, Gonzalez B, Campbell IL, Hidalgo J. Localization of metallothionein-I and -III expression in the CNS of transgenic mice with astrocyte-targeted expression of interleukin 6. Exp Neurol. 1998;153:184–94. 9784278 10.1006/exnr.1998.6861

[18] Cerrato V. Cerebellar astrocytes: much more than passive bystanders in ataxia pathophysiology. J Clin Med. 2020;9:757. 32168822 10.3390/jcm9030757 PMC7141261

[19] Chiang CS, Stalder A, Samimi A, Campbell IL. Reactive gliosis as a consequence of interleukin-6 expression in the brain: studies in transgenic mice. Dev Neurosci. 1994;16:212–21. 7535683 10.1159/000112109

[20] Chibowska K, Korbecki J, Gutowska I, Metryka E, Tarnowski M, Goschorska M, Barczak K, Chlubek D, Baranowska-Bosiacka I. Pre- and neonatal exposure to Lead (Pb) induces neuroinflammation in the forebrain cortex, hippocampus and cerebellum of rat pups. Int J Mol Sci. 2020;21. 10.3390/ijms21031083 PMC7037720 32041252

[21] Chung WS, Allen NJ, Eroglu C. Astrocytes control synapse formation function, and elimination. Cold Spring Harbor Perspect Biol. 2015;7:a020370. 10.1101/cshperspect.a020370 PMC4527946 25663667

[22] Colonna M, Butovsky O. Microglia function in the central nervous system during health and neurodegeneration. Annu Rev Immunol. 2017;35:441–68. 28226226 10.1146/annurev-immunol-051116-052358 PMC8167938

[23] Conroy SM, Nguyen V, Quina LA, Blakely-Gonzales P, Ur C, Netzeband JG, Prieto AL, Gruol DL. Interleukin-6 produces neuronal loss in developing cerebellar granule neuron cultures. J Neuroimmunol. 2004;155:43–54. 15342195 10.1016/j.jneuroim.2004.06.014

[24] Corbin JG, Kelly D, Rath EM, Baerwald KD, Suzuki K, Popko B. Targeted CNS expression of interferon-gamma in transgenic mice leads to hypomyelination, reactive gliosis, and abnormal cerebellar development. Mol Cell Neurosci. 1996;7:354–70. 8812062 10.1006/mcne.1996.0026

[25] Cornell J, Salinas S, Huang HY, Zhou M. Microglia regulation of synaptic plasticity and learning and memory. Neural Regen Res. 2022;17:705–16. 34472455 10.4103/1673-5374.322423 PMC8530121

[26] Cunningham C, Dunne A, Lopez-Rodriguez AB. Astrocytes: Heterogeneous and dynamic phenotypes in neurodegeneration and innate immunity. Neuroscientist : Rev J Bringing Neurobiol, Neurol Psychiatry. 2019;25:455–74. 10.1177/1073858418809941 PMC6525076 30451065

[27] Cvetanovic M, Ingram M, Orr H, Opal P. Early activation of microglia and astrocytes in mouse models of spinocerebellar ataxia type 1. Neuroscience. 2015;289:289–99. 25595967 10.1016/j.neuroscience.2015.01.003 PMC4344857

[28] Dinarello CA. Historical insights into cytokines. Eur J Immunol. 2007;37(Suppl 1):S34-45. 17972343 10.1002/eji.200737772 PMC3140102

[29] Dos Santos SE, Medeiros M, Porfirio J, Tavares W, Pessôa L, Grinberg L, Leite REP, Ferretti-Rebustini REL, Suemoto CK, Filho WJ, Noctor SC, Sherwood CC, Kaas JH, Manger PR, Herculano-Houzel S. Similar microglial cell densities across brain structures and mammalian species: implications for brain tissue function. J Neurosci: Off J Soc Neurosci. 2020;40:4622–43. 10.1523/JNEUROSCI.2339-19.2020 PMC7294795 32253358

[30] Dufour BD, Amina S, Martinez-Cerdeno V. FXTAS presents with upregulation of the cytokines IL12 and TNFα. Parkinsonism Relat Disord. 2021;82:117–20. 33285358 10.1016/j.parkreldis.2020.11.026 PMC8070980

[31] Elvers M, Pfeiffer J, Kaltschmidt C, Kaltschmidt B. TGF-beta2 neutralization inhibits proliferation and activates apoptosis of cerebellar granule cell precurors in the developing cerebellum. Mech Dev. 2005;122:587–602. 15804570 10.1016/j.mod.2004.10.012

[32] Emsley JG, Macklis JD. Astroglial heterogeneity closely reflects the neuronal-defined anatomy of the adult murine CNS. Neuron Glia Biol. 2006;2:175–86. 17356684 10.1017/S1740925X06000202 PMC1820889

[33] Escartin C, Galea E, Lakatos A, O’Callaghan JP, Petzold GC, Serrano-Pozo A, Steinhäuser C, Volterra A, Carmignoto G, Agarwal A, Allen NJ, Araque A, Barbeito L, Barzilai A, Bergles DE, Bonvento G, Butt AM, Chen WT, Cohen-Salmon M, Cunningham C, Deneen B, De Strooper B, Díaz-Castro B, Farina C, Freeman M, Gallo V, Goldman JE, Goldman SA, Götz M, Gutiérrez A, Haydon PG, Heiland DH, Hol EM, Holt MG, Iino M, Kastanenka KV, Kettenmann H, Khakh BS, Koizumi S, Lee CJ, Liddelow SA, MacVicar BA, Magistretti P, Messing A, Mishra A, Molofsky AV, Murai KK, Norris CM, Okada S, Oliet SHR, Oliveira JF, Panatier A, Parpura V, Pekna M, Pekny M, Pellerin L, Perea G, Pérez-Nievas BG, Pfrieger FW, Poskanzer KE, Quintana FJ, Ransohoff RM, Riquelme-Perez M, Robel S, Rose CR, Rothstein JD, Rouach N, Rowitch DH, Semyanov A, Sirko S, Sontheimer H, Swanson RA, Vitorica J, Wanner IB, Wood LB, Wu J, Zheng B, Zimmer ER, Zorec R, Sofroniew MV, Verkhratsky A. Reactive astrocyte nomenclature, definitions, and future directions. Nat Neurosci. 2021;24:312–25. 33589835 10.1038/s41593-020-00783-4 PMC8007081

[34] Fattori E, Lazzaro D, Musiani P, Modesti A, Alonzi T, Ciliberto G. IL-6 expression in neurons of transgenic mice causes reactive astrocytosis and increase in ramified microglial cells but no neuronal damage. Eur J Neurosci. 1995;7:2441–9. 8845949 10.1111/j.1460-9568.1995.tb01042.x

[35] Ferro A, Sheeler C, Rosa JG, Cvetanovic M. Role of Microglia in Ataxias. J Mol Biol. 2019;431:1792–804. 30660620 10.1016/j.jmb.2019.01.016 PMC7164490

[36] Fiacco TA, McCarthy KD. Astrocyte calcium elevations: properties, propagation, and effects on brain signaling. Glia. 2006;54:676–90. 17006896 10.1002/glia.20396

[37] Fraunberger EA, DeJesus P, Zanier ER, Shutt TE, Esser MJ. Acute and persistent alterations of cerebellar inflammatory networks and glial activation in a rat model of pediatric mild traumatic brain injury. J Neurotrauma. 2020;37:1315–30. 31808365 10.1089/neu.2019.6714

[38] French RA, VanHoy RW, Chizzonite R, Zachary JF, Dantzer R, Parnet P, Bluthé RM, Kelley KW. Expression and localization of p80 and p68 interleukin-1 receptor proteins in the brain of adult mice. J Neuroimmunol. 1999;93:194–202. 10378883 10.1016/s0165-5728(98)00224-0

[39] García-Juárez M, Camacho-Morales A. Defining the role of anti- and pro-inflammatory outcomes of interleukin-6 in mental health. Neuroscience. 2022;492:32–46. 35439579 10.1016/j.neuroscience.2022.03.020

[40] Garden GA. Microglia in human immunodeficiency virus-associated neurodegeneration. Glia. 2002;40:240–51. 12379911 10.1002/glia.10155

[41] Garland EF, Hartnell IJ, Boche D. Microglia and astrocyte function and communication: what do we know in humans? Front Neurosci. 2022;16:824888. 35250459 10.3389/fnins.2022.824888 PMC8888691

[42] Giovannelli A, Limatola C, Ragozzino D, Mileo AM, Ruggieri A, Ciotti MT, Mercanti D, Santoni A, Eusebi F. CXC chemokines interleukin-8 (IL-8) and growth-related gene product alpha (GROalpha) modulate Purkinje neuron activity in mouse cerebellum. J Neuroimmunol. 1998;92:122–32. 9916887 10.1016/s0165-5728(98)00192-1

[43] Goenaga J, Araque A, Kofuji P, Herrera Moro Chao D. Calcium signaling in astrocytes and gliotransmitter release. Front Synaptic Neurosci. 2023;15:1138577. 36937570 10.3389/fnsyn.2023.1138577 PMC10017551

[44] Gotoh M, Miyamoto Y, Ikeshima-Kataoka H. Astrocytic neuroimmunological roles interacting with microglial cells in neurodegenerative diseases. Int J Mol Sci. 2023;24:1599. 36675113 10.3390/ijms24021599 PMC9865248

[45] Grabert K, Michoel T, Karavolos MH, Clohisey S, Baillie JK, Stevens MP, Freeman TC, Summers KM, McColl BW. Microglial brain region-dependent diversity and selective regional sensitivities to aging. Nat Neurosci. 2016;19:504–16. 26780511 10.1038/nn.4222 PMC4768346

[46] Gruol DL. Impact of increased astrocyte expression of IL-6, CCL2 or CXCL10 in transgenic mice on hippocampal synaptic function. Brain Sci. 2016;6:19. 27322336 10.3390/brainsci6020019 PMC4931496

[47] Gruol DL, Vo K, Bray JG. Increased astrocyte expression of IL-6 or CCL2 in transgenic mice alters levels of hippocampal and cerebellar proteins. Front Cell Neurosci. 2014;8:234. 25177271 10.3389/fncel.2014.00234 PMC4132577

[48] Gyengesi E, Rangel A, Ullah F, Liang H, Niedermayer G, Asgarov R, Venigalla M, Gunawardena D, Karl T, Munch G. Chronic microglial activation in the GFAP-IL6 mouse contributes to age-dependent cerebellar volume loss and impairment in motor function. Front Neurosci. 2019;13:303. 31001075 10.3389/fnins.2019.00303 PMC6456818

[49] Heaney ML, Golde DW. Soluble cytokine receptors. Blood. 1996;87:847–57. 8562952

[50] Hickman S, Izzy S, Sen P, Morsett L, El Khoury J. Microglia in neurodegeneration. Nat Neurosci. 2018;21:1359–69. 30258234 10.1038/s41593-018-0242-x PMC6817969

[51] Holloway KN, Pinson MR, Douglas JC, Rafferty TM, Kane CJM, Miranda RC, Drew PD. Cerebellar transcriptomic analysis in a chronic plus binge mouse model of alcohol use disorder demonstrates ethanol-induced neuroinflammation and altered glial gene expression. Cells. 2023;12:745. 36899881 10.3390/cells12050745 PMC10000476

[52] Hoogland TM, Kuhn B. Recent developments in the understanding of astrocyte function in the cerebellum in vivo. Cerebellum. 2010;9:264–71. 19904577 10.1007/s12311-009-0139-z

[53] Hoogland TM, Kuhn B, Göbel W, Huang W, Nakai J, Helmchen F, Flint J, Wang SS. Radially expanding transglial calcium waves in the intact cerebellum. Proc Natl Acad Sci USA. 2009;106:3496–501. 19211787 10.1073/pnas.0809269106 PMC2651231

[54] Ishijima T, Nakajima K. Mechanisms of microglia proliferation in a rat model of facial nerve anatomy. Biology (Basel). 2023;12:1121. 37627005 10.3390/biology12081121 PMC10452325

[55] Jha MK, Jo M, Kim JH, Suk K. Microglia-astrocyte crosstalk: an intimate molecular conversation. Neuroscientist : Rev J Bringing Neurobiol, Neurol Psychiatry. 2019;25:227–40. 10.1177/1073858418783959 29931997

[56] Jha MK, Kim JH, Song GJ, Lee WH, Lee IK, Lee HW, An SSA, Kim S, Suk K. Functional dissection of astrocyte-secreted proteins: implications in brain health and diseases. Prog Neurobiol. 2018;162:37–69. 29247683 10.1016/j.pneurobio.2017.12.003

[57] John Lin CC, Yu K, Hatcher A, Huang TW, Lee HK, Carlson J, Weston MC, Chen F, Zhang Y, Zhu W, Mohila CA, Ahmed N, Patel AJ, Arenkiel BR, Noebels JL, Creighton CJ, Deneen B. Identification of diverse astrocyte populations and their malignant analogs. Nat Neurosci. 2017;20:396–405. 28166219 10.1038/nn.4493 PMC5824716

[58] Kana V, Desland FA, Casanova-Acebes M, Ayata P, Badimon A, Nabel E, Yamamuro K, Sneeboer M, Tan IL, Flanigan ME, Rose SA, Chang C, Leader A, Le Bourhis H, Sweet ES, Tung N, Wroblewska A, Lavin Y, See P, Baccarini A, Ginhoux F, Chitu V, Stanley ER, Russo SJ, Yue Z, Brown BD, Joyner AL, De Witte LD, Morishita H, Schaefer A, Merad M. CSF-1 controls cerebellar microglia and is required for motor function and social interaction. J Exp Med. 2019;216:2265–81. 31350310 10.1084/jem.20182037 PMC6781012

[59] Kane CJ, Phelan KD, Douglas JC, Wagoner G, Johnson JW, Xu J, Phelan PS, Drew PD. Effects of ethanol on immune response in the brain: region-specific changes in adolescent versus adult mice. Alcohol Clin Exp Res. 2014;38:384–91. 24033454 10.1111/acer.12244 PMC3872252

[60] Kaul M, Lipton SA. Mechanisms of neuroimmunity and neurodegeneration associated with HIV-1 infection and AIDS. J Neuroimmune Pharmacol. 2006;1:138–51. 18040780 10.1007/s11481-006-9011-9

[61] Kaur C, Sivakumar V, Zou Z, Ling EA. Microglia-derived proinflammatory cytokines tumor necrosis factor-alpha and interleukin-1beta induce Purkinje neuronal apoptosis via their receptors in hypoxic neonatal rat brain. Brain Struct Funct. 2014;219:151–70. 23262920 10.1007/s00429-012-0491-5

[62] Kempuraj D, Thangavel R, Natteru PA, Selvakumar GP, Saeed D, Zahoor H, Zaheer S, Iyer SS, Zaheer A. Neuroinflammation induces neurodegeneration. J Neurol Neurosurg Spine. 2016;1:1003. 28127589 PMC5260818

[63] Kimelberg HK. Functions of mature mammalian astrocytes: a current view. Neuroscientist : Rev J Bringing Neurobiol, Neurol Psychiatry. 2010;16:79–106. 10.1177/1073858409342593 20236950

[64] Kozuki M, Kurata T, Miyazaki K, Morimoto N, Ohta Y, Ikeda Y, Abe K. Atorvastatin and pitavastatin protect cerebellar Purkinje cells in AD model mice and preserve the cytokines MCP-1 and TNF-α. Brain Res. 2011;1388:32–8. 21419111 10.1016/j.brainres.2011.03.024

[65] LaFerla FM, Sugarman MC, Lane TE, Leissring MA. Regional hypomyelination and dysplasia in transgenic mice with astrocyte-directed expression of interferon-gamma. J Mol Neurosci. 2000;15:45–59. 11211236 10.1385/JMN:15:1:45

[66] Lehre KP, Danbolt NC. The number of glutamate transporter subtype molecules at glutamatergic synapses: chemical and stereological quantification in young adult rat brain, The Journal of neuroscience : the official journal of the Society for. Neuroscience. 1998;18:8751–7. 9786982 10.1523/JNEUROSCI.18-21-08751.1998 PMC6793562

[67] Levi H, Bar E, Cohen-Adiv S, Sweitat S, Kanner S, Galron R, Mitiagin Y, Barzilai A. Dysfunction of cerebellar microglia in Ataxia-telangiectasia. Glia. 2022;70:536–57. 34854502 10.1002/glia.24122

[68] Levine SJ. Mechanisms of soluble cytokine receptor generation. J Immunol. 2004;173:5343–8. 15494479 10.4049/jimmunol.173.9.5343

[69] Li L, Acioglu C, Heary RF, Elkabes S. Role of astroglial toll-like receptors (TLRs) in central nervous system infections, injury and neurodegenerative diseases. Brain Behav Immun. 2021;91:740–55. 33039660 10.1016/j.bbi.2020.10.007 PMC7543714

[70] Li Q, Barres BA. Microglia and macrophages in brain homeostasis and disease. Nat Rev Immunol. 2018;18:225–42. 29151590 10.1038/nri.2017.125

[71] Limatola C, Giovannelli A, Maggi L, Ragozzino D, Castellani L, Ciotti MT, Vacca F, Mercanti D, Santoni A, Eusebi F. SDF-1alpha-mediated modulation of synaptic transmission in rat cerebellum. Eur J Neurosci. 2000;12:2497–504. 10947825 10.1046/j.1460-9568.2000.00139.x

[72] Lin W, Kemper A, McCarthy KD, Pytel P, Wang JP, Campbell IL, Utset MF, Popko B. Interferon-gamma induced medulloblastoma in the developing cerebellum, The Journal of neuroscience : the official journal of the Society for. Neuroscience. 2004;24:10074–83. 15537876 10.1523/JNEUROSCI.2604-04.2004 PMC6730177

[73] Liu C, Chu D, Kalantar-Zadeh K, George J, Young HA, Liu G. Cytokines: from clinical significance to quantification. Adv Sci (Weinh). 2021;8:e2004433. 34114369 10.1002/advs.202004433 PMC8336501

[74] Llorens F, López-González I, Thüne K, Carmona M, Zafar S, Andréoletti O, Zerr I, Ferrer I. Subtype and regional-specific neuroinflammation in sporadic creutzfeldt-jakob disease. Front Aging Neurosci. 2014;6:198. 25136317 10.3389/fnagi.2014.00198 PMC4120692

[75] Lokau J, Garbers C. Biological functions and therapeutic opportunities of soluble cytokine receptors. Cytokine Growth Factor Rev. 2020;55:94–108. 32386776 10.1016/j.cytogfr.2020.04.003

[76] Luan J, Furuta Y, Du J, Richmond A. Developmental expression of two CXC chemokines, MIP-2 and KC, and their receptors. Cytokine. 2001;14:253–63. 11444905 10.1006/cyto.2001.0882 PMC5433622

[77] Mandolesi G, Musella A, Gentile A, Grasselli G, Haji N, Sepman H, Fresegna D, Bullitta S, De Vito F, Musumeci G, Di Sanza C, Strata P, Centonze D. Interleukin-1β alters glutamate transmission at purkinje cell synapses in a mouse model of multiple sclerosis. J Neurosci: Off J Soc Neurosci. 2013;33:12105–21. 10.1523/JNEUROSCI.5369-12.2013 PMC6794065 23864696

[78] Metea MR, Newman EA. Calcium signaling in specialized glial cells. Glia. 2006;54:650–5. 17006893 10.1002/glia.20352 PMC2289783

[79] Morikawa Y, Tohya K, Tamura S, Ichihara M, Miyajima A, Senba E. Expression of interleukin-6 receptor, leukemia inhibitory factor receptor and glycoprotein 130 in the murine cerebellum and neuropathological effect of leukemia inhibitory factor on cerebellar Purkinje cells. Neuroscience. 2000;100:841–8. 11036218 10.1016/s0306-4522(00)00302-x

[80] Motoki K, Kishi H, Hori E, Tajiri K, Nishijo H, Muraguchi A. The direct excitatory effect of IL-1beta on cerebellar Purkinje cell. Biochem Biophys Res Commun. 2009;379:665–8. 19100239 10.1016/j.bbrc.2008.12.023

[81] Mühleisen H, Gehrmann J, Meyermann R. Reactive microglia in Creutzfeldt-Jakob disease. Neuropathol Appl Neurobiol. 1995;21:505–17. 8745240 10.1111/j.1365-2990.1995.tb01097.x

[82] Nakayama H, Abe M, Morimoto C, Iida T, Okabe S, Sakimura K, Hashimoto K. Microglia permit climbing fiber elimination by promoting GABAergic inhibition in the developing cerebellum. Nat Commun. 2018;9:2830. 30026565 10.1038/s41467-018-05100-z PMC6053401

[83] Nelson TE, Campbell IL, Gruol DL. Altered physiology of Purkinje neurons in cerebellar slices from transgenic mice with chronic central nervous system expression of interleukin-6. Neuroscience. 1999;89:127–36. 10051222 10.1016/s0306-4522(98)00316-9

[84] Nelson TE, Netzeband JG, Gruol DL. Chronic interleukin-6 exposure alters metabotropic glutamate receptor-activated calcium signalling in cerebellar Purkinje neurons. Eur J Neurosci. 2004;20:2387–400. 15525280 10.1111/j.1460-9568.2004.03706.x

[85] Nelson TE, Ur CL, Gruol DL. Chronic interleukin-6 exposure alters electrophysiological properties and calcium signaling in developing cerebellar purkinje neurons in culture. J Neurophysiol. 2002;88:475–86. 12091569 10.1152/jn.2002.88.1.475

[86] Niedzwiedz-Massey VM, Douglas JC, Rafferty T, Johnson JW, Holloway KN, Berquist MD, Kane CJM, Drew PD. Effects of chronic and binge ethanol administration on mouse cerebellar and hippocampal neuroinflammation. Am J Drug Alcohol Abuse. 2022;49:345–58. 36345683 10.1080/00952990.2022.2128361 PMC10615135

[87] Nimmerjahn A, Kirchhoff F, Helmchen F. Resting microglial cells are highly dynamic surveillants of brain parenchyma in vivo. Science (New York, NY). 2005;308:1314–8. 10.1126/science.1110647 15831717

[88] Nottet HS, Gendelman HE. Unraveling the neuroimmune mechanisms for the HIV-1-associated cognitive/motor complex. Immunol Today. 1995;16:441–8. 7546209 10.1016/0167-5699(95)80022-0

[89] Okun E, Griffioen KJ, Mattson MP. Toll-like receptor signaling in neural plasticity and disease. Trends Neurosci. 2011;34:269–81. 21419501 10.1016/j.tins.2011.02.005 PMC3095763

[90] Oprica M, Hjorth E, Spulber S, Popescu BO, Ankarcrona M, Winblad B, Schultzberg M. Studies on brain volume, Alzheimer-related proteins and cytokines in mice with chronic overexpression of IL-1 receptor antagonist. J Cell Mol Med. 2007;11:810–25. 17760842 10.1111/j.1582-4934.2007.00074.x PMC3823259

[91] Ozawa PM, Ariza CB, Ishibashi CM, Fujita TC, Banin-Hirata BK, Oda JM, Watanabe MA. Role of CXCL12 and CXCR4 in normal cerebellar development and medulloblastoma. Int J Cancer. 2016;138:10–3. 25400097 10.1002/ijc.29333

[92] Palomo J, Dietrich D, Martin P, Palmer G, Gabay C. The interleukin (IL)-1 cytokine family–Balance between agonists and antagonists in inflammatory diseases. Cytokine. 2015;76:25–37. 26185894 10.1016/j.cyto.2015.06.017

[93] Pannasch U, Rouach N. Emerging role for astroglial networks in information processing: from synapse to behavior. Trends Neurosci. 2013;36:405–17. 23659852 10.1016/j.tins.2013.04.004

[94] Paolicelli RC, Sierra A, Stevens B, Tremblay ME, Aguzzi A, Ajami B, Amit I, Audinat E, Bechmann I, Bennett M, Bennett F, Bessis A, Biber K, Bilbo S, Blurton-Jones M, Boddeke E, Brites D, Brône B, Brown GC, Butovsky O, Carson MJ, Castellano B, Colonna M, Cowley SA, Cunningham C, Davalos D, De Jager PL, de Strooper B, Denes A, Eggen BJL, Eyo U, Galea E, Garel S, Ginhoux F, Glass CK, Gokce O, Gomez-Nicola D, González B, Gordon S, Graeber MB, Greenhalgh AD, Gressens P, Greter M, Gutmann DH, Haass C, Heneka MT, Heppner FL, Hong S, Hume DA, Jung S, Kettenmann H, Kipnis J, Koyama R, Lemke G, Lynch M, Majewska A, Malcangio M, Malm T, Mancuso R, Masuda T, Matteoli M, McColl BW, Miron VE, Molofsky AV, Monje M, Mracsko E, Nadjar A, Neher JJ, Neniskyte U, Neumann H, Noda M, Peng B, Peri F, Perry VH, Popovich PG, Pridans C, Priller J, Prinz M, Ragozzino D, Ransohoff RM, Salter MW, Schaefer A, Schafer DP, Schwartz M, Simons M, Smith CJ, Streit WJ, Tay TL, Tsai LH, Verkhratsky A, von Bernhardi R, Wake H, Wittamer V, Wolf SA, Wu LJ, Wyss-Coray T. Microglia states and nomenclature: a field at its crossroads. Neuron. 2022;110:3458–83. 36327895 10.1016/j.neuron.2022.10.020 PMC9999291

[95] Park CR, Kim DK, Cho EB, You DJ, do Rego JL, Vaudry D, Sun W, Kim H, Seong JY, Hwang JI. Spatiotemporal expression and functional implication of CXCL14 in the developing mice cerebellum. Mol Cells. 2012;34:289–93. 22843118 10.1007/s10059-012-0116-0 PMC3887834

[96] Paschon V, Takada SH, Ikebara JM, Sousa E, Raeisossadati R, Ulrich H, Kihara AH. Interplay between exosomes, microRNAs and toll-like receptors in brain disorders. Mol Neurobiol. 2016;53:2016–28. 25862375 10.1007/s12035-015-9142-1

[97] Peng YP, Qiu YH, Lu JH, Wang JJ. Interleukin-6 protects cultured cerebellar granule neurons against glutamate-induced neurotoxicity. Neurosci Lett. 2005;374:192–6. 15663961 10.1016/j.neulet.2004.10.069

[98] Potts MB, Adwanikar H, Noble-Haeusslein LJ. Models of traumatic cerebellar injury. Cerebellum. 2009;8:211–21. 19495901 10.1007/s12311-009-0114-8 PMC2734258

[99] Prinz M, Priller J, Sisodia SS, Ransohoff RM. Heterogeneity of CNS myeloid cells and their roles in neurodegeneration. Nat Neurosci. 2011;14:1227–35. 21952260 10.1038/nn.2923

[100] Probert L, Akassoglou K, Kassiotis G, Pasparakis M, Alexopoulou L, Kollias G. TNF-alpha transgenic and knockout models of CNS inflammation and degeneration. J Neuroimmunol. 1997;72:137–41. 9042105 10.1016/s0165-5728(96)00184-1

[101] Qiu Z, Sweeney DD, Netzeband JG, Gruol DL. Chronic interleukin-6 alters NMDA receptor-mediated membrane responses and enhances neurotoxicity in developing CNS neurons. J Neurosci: Off J Soc Neurosci. 1998;18:10445–56. 10.1523/JNEUROSCI.18-24-10445.1998 PMC6793367 9852582

[102] Qu W, Johnson A, Kim JH, Lukowicz A, Svedberg D, Cvetanovic M. Inhibition of colony-stimulating factor 1 receptor early in disease ameliorates motor deficits in SCA1 mice. J Neuroinflammation. 2017;14:107. 28545543 10.1186/s12974-017-0880-z PMC5445366

[103] Ragozzino D. CXC chemokine receptors in the central nervous system: Role in cerebellar neuromodulation and development. J Neurovirol. 2002;8:559–72. 12476350 10.1080/13550280290100932

[104] Ragozzino D, Giovannelli A, Mileo AM, Limatola C, Santoni A, Eusebi F. Modulation of the neurotransmitter release in rat cerebellar neurons by GRO beta. NeuroReport. 1998;9:3601–6. 9858367 10.1097/00001756-199811160-00011

[105] Rasmussen CLM, Thomsen LB, Heegaard CW, Moos T, Burkhart A. The Npc2(Gt(LST105)BygNya) mouse signifies pathological changes comparable to human Niemann-Pick type C2 disease. Mol Cell Neurosci. 2023;126:103880. 37454976 10.1016/j.mcn.2023.103880

[106] Revuelta M, Scheuer T, Chew LJ, Schmitz T. Glial factors regulating white matter development and pathologies of the cerebellum. Neurochem Res. 2020;45:643–55. 31974933 10.1007/s11064-020-02961-z PMC7058568

[107] Rose-John S. IL-6 trans-signaling via the soluble IL-6 receptor: importance for the pro-inflammatory activities of IL-6. Int J Biol Sci. 2012;8:1237–47. 23136552 10.7150/ijbs.4989 PMC3491447

[108] Rose-John S, Scheller J, Elson G, Jones SA. Interleukin-6 biology is coordinated by membrane-bound and soluble receptors: role in inflammation and cancer. J Leukoc Biol. 2006;80:227–36. 16707558 10.1189/jlb.1105674

[109] Saberi D, Ott B, Dahlke C, Matschke V, Schmitt-John T, Theiss C. The spatiotemporal pattern of degeneration in the cerebellum of the wobbler mouse. J Neuropathol Exp Neurol. 2016;75:347–57. 26945034 10.1093/jnen/nlw005

[110] Sahbaz NA, Peker KD, Kabuli HA, Gumusoglu AY, Alis H. Single center experience in laparoscopic treatment of gallbladder perforation. Wideochirurgia i inne techniki maloinwazyjne = Videosurgery Other Miniinvasive Tech. 2017;12:372–7. 10.5114/wiitm.2017.72321 PMC5776488 29362652

[111] Sauder C, de la Torre JC. Cytokine expression in the rat central nervous system following perinatal Borna disease virus infection. J Neuroimmunol. 1999;96:29–45. 10227422 10.1016/s0165-5728(98)00272-0

[112] Saxton RA, Glassman CR, Garcia KC. Emerging principles of cytokine pharmacology and therapeutics. Nat Rev Drug Discov. 2023;22:21–37. 36131080 10.1038/s41573-022-00557-6 PMC10292932

[113] Scheller J, Chalaris A, Schmidt-Arras D, Rose-John S. The pro- and anti-inflammatory properties of the cytokine interleukin-6. Biochem Biophys Acta. 2011;1813:878–88. 21296109 10.1016/j.bbamcr.2011.01.034

[114] Schmahmann JD. The cerebellum and cognition. Neurosci Lett. 2019;688:62–75. 29997061 10.1016/j.neulet.2018.07.005

[115] Schmahmann JD. Emotional disorders and the cerebellum: Neurobiological substrates, neuropsychiatry, and therapeutic implications. Handb Clin Neurol. 2021;183:109–54. 34389114 10.1016/B978-0-12-822290-4.00016-5

[116] Schwartz DM, Bonelli M, Gadina M, O’Shea JJ. Type I/II cytokines, JAKs, and new strategies for treating autoimmune diseases. Nat Rev Rheumatol. 2016;12:25–36. 26633291 10.1038/nrrheum.2015.167 PMC4688091

[117] Shim HG, Jang SS, Kim SH, Hwang EM, Min JO, Kim HY, Kim YS, Ryu C, Chung G, Kim Y, Yoon BE, Kim SJ. TNF-α increases the intrinsic excitability of cerebellar Purkinje cells through elevating glutamate release in Bergmann Glia. Sci Rep. 2018;8:11589. 30072733 10.1038/s41598-018-29786-9 PMC6072779

[118] Singh-Bains MK, Linke V, Austria MDR, Tan AYS, Scotter EL, Mehrabi NF, Faull RLM, Dragunow M. Altered microglia and neurovasculature in the Alzheimer’s disease cerebellum. Neurobiol Dis. 2019;132:104589. 31454549 10.1016/j.nbd.2019.104589

[119] Smith JA, Das A, Ray SK, Banik NL. Role of pro-inflammatory cytokines released from microglia in neurodegenerative diseases. Brain Res Bull. 2012;87:10–20. 22024597 10.1016/j.brainresbull.2011.10.004 PMC9827422

[120] Stephenson J, Nutma E, van der Valk P, Amor S. Inflammation in CNS neurodegenerative diseases. Immunology. 2018;154:204–19. 29513402 10.1111/imm.12922 PMC5980185

[121] Stoessel MB, Majewska AK. Little cells of the little brain: microglia in cerebellar development and function. Trends Neurosci. 2021;44:564–78. 33933255 10.1016/j.tins.2021.04.001 PMC8222145

[122] Stowell RD, Wong EL, Batchelor HN, Mendes MS, Lamantia CE, Whitelaw BS, Majewska AK. Cerebellar microglia are dynamically unique and survey Purkinje neurons in vivo. Dev Neurobiol. 2018;78:627–44. 29285893 10.1002/dneu.22572 PMC6544048

[123] Su X, Guan W, Yu YC, Fu Y. Cerebellar stem cells do not produce neurons and astrocytes in adult mouse. Biochem Biophys Res Commun. 2014;450:378–83. 24944019 10.1016/j.bbrc.2014.05.131

[124] Subramaniam SR, Federoff HJ. Targeting microglial activation states as a therapeutic avenue in Parkinson’s disease. Front Aging Neurosci. 2017;9:176. 28642697 10.3389/fnagi.2017.00176 PMC5463358

[125] Sun XM, Lu JH, Qiu YH, Liu Z, Wang XQ, Peng YP. Interleukin-6 reduces NMDA-induced Ca2+ overload via prevention of Ca2+ release from intracellular store. Int J Neurosci. 2011;121:423–9. 21781004 10.3109/00207454.2011.556280

[126] Tan YL, Yuan Y, Tian L. Microglial regional heterogeneity and its role in the brain. Mol Psychiatry. 2020;25:351–67. 31772305 10.1038/s41380-019-0609-8 PMC6974435

[127] Thomas Curtis J, Chen Y, Buck DJ, Davis RL. Chronic inorganic mercury exposure induces sex-specific changes in central TNFα expression: importance in autism. Neurosci Lett. 2011;504:40–4. 21906657 10.1016/j.neulet.2011.08.053 PMC3443965

[128] Tiveron MC, Cremer H. CXCL12/CXCR4 signalling in neuronal cell migration. Curr Opin Neurobiol. 2008;18:237–44. 18644448 10.1016/j.conb.2008.06.004

[129] Tomita M, Holman BJ, Williams LS, Pang KC, Santoro TJ. Cerebellar dysfunction is associated with overexpression of proinflammatory cytokine genes in lupus. J Neurosci Res. 2001;64:26–33. 11276048 10.1002/jnr.1050

[130] Topper LA, Baculis BC, Valenzuela CF. Exposure of neonatal rats to alcohol has differential effects on neuroinflammation and neuronal survival in the cerebellum and hippocampus. J Neuroinflammation. 2015;12:160. 26337952 10.1186/s12974-015-0382-9 PMC4558631

[131] Umpierre AD, Wu LJ. How microglia sense and regulate neuronal activity. Glia. 2021;69:1637–53. 33369790 10.1002/glia.23961 PMC8113084

[132] UriarteHuarte O, Richart L, Mittelbronn M, Michelucci A. Microglia in Health and Disease: The Strength to Be Diverse and Reactive. Front Cell Neurosci. 2021;15:660523. 33867943 10.3389/fncel.2021.660523 PMC8044310

[133] Vainchtein ID, Molofsky AV. Astrocytes and microglia in sickness and in health. Trends Neurosci. 2020;43:144–54. 32044129 10.1016/j.tins.2020.01.003 PMC7472912

[134] van Gassen KL, Netzeband JG, de Graan PN, Gruol DL. The chemokine CCL2 modulates Ca2+ dynamics and electrophysiological properties of cultured cerebellar Purkinje neurons. Eur J Neurosci. 2005;21:2949–57. 15978006 10.1111/j.1460-9568.2005.04113.x

[135] Vargas DL, Nascimbene C, Krishnan C, Zimmerman AW, Pardo CA. Neuroglial activation and neuroinflammation in the brain of patients with autism. Ann Neurol. 2005;57:67–81. 15546155 10.1002/ana.20315

[136] Verkhratsky A. Glial calcium signaling in physiology and pathophysiology. Acta Pharmacol Sin. 2006;27:773–80. 16787559 10.1111/j.1745-7254.2006.00396.x

[137] Verkhratsky A, Nedergaard M. Physiology of astroglia. Physiol Rev. 2018;98:239–389. 29351512 10.1152/physrev.00042.2016 PMC6050349

[138] Verkhratsky A, Rodríguez JJ, Steardo L. Astrogliopathology: a central element of neuropsychiatric diseases? Neuroscientist : Rev J Bringing Neurobiol, Neurol Psychiatry. 2014;20:576–88. 10.1177/1073858413510208 24301046

[139] Vernet-der Garabedian B, Lemaigre-Dubreuil Y, Delhaye-Bouchaud N, Mariani J. Abnormal IL-1beta cytokine expression in the cerebellum of the ataxic mutant mice staggerer and lurcher. Brain Res Mol Brain Res. 1998;62:224–7. 9813341 10.1016/s0169-328x(98)00268-x

[140] Vlassaks E, Brudek T, Pakkenberg B, Gavilanes AW. Cerebellar cytokine expression in a rat model for fetal asphyctic preconditioning and perinatal asphyxia. Cerebellum. 2014;13:471–8. 24771476 10.1007/s12311-014-0559-2 PMC4076859

[141] von Bartheld CS, Bahney J, Herculano-Houzel S. The search for true numbers of neurons and glial cells in the human brain: a review of 150 years of cell counting. J Comp Neurol. 2016;524:3865–95. 27187682 10.1002/cne.24040 PMC5063692

[142] Wang J, Pham-Mitchell N, Schindler C, Campbell IL. Dysregulated Sonic hedgehog signaling and medulloblastoma consequent to IFN-alpha-stimulated STAT2-independent production of IFN-gamma in the brain. J Clin Invest. 2003;112:535–43. 12925694 10.1172/JCI18637 PMC171394

[143] Wei H, Zou H, Sheikh AM, Malik M, Dobkin C, Brown WT, Li X. IL-6 is increased in the cerebellum of autistic brain and alters neural cell adhesion, migration and synaptic formation. J Neuroinflammation. 2011;8:52. 21595886 10.1186/1742-2094-8-52 PMC3114764

[144] Woodburn SC, Bollinger JL, Wohleb ES. The semantics of microglia activation: neuroinflammation, homeostasis, and stress. J Neuroinflammation. 2021;18:258. 34742308 10.1186/s12974-021-02309-6 PMC8571840

[145] Xie ST, Fan WC, Zhao XS, Ma XY, Li ZL, Zhao YR, Yang F, Shi Y, Rong H, Cui ZS, Chen JY, Li HZ, Yan C, Zhang Q, Wang JJ, Zhang XY, Gu XP, Ma ZL, Zhu JN. Proinflammatory activation of microglia in the cerebellum hyperexcites Purkinje cells to trigger ataxia. Pharmacol Res. 2023;191. 10.1016/j.phrs.2023.106773 37068531

[146] Yamamoto M, Kim M, Imai H, Itakura Y, Ohtsuki G. Microglia-triggered plasticity of intrinsic excitability modulates psychomotor behaviors in acute cerebellar inflammation. Cell Rep. 2019;28:2923-2938.e8. 31509752 10.1016/j.celrep.2019.07.078

[147] Yoshioka M, Bradley WG, Shapshak P, Nagano I, Stewart RV, Xin KQ, Srivastava AK, Nakamura S. Role of immune activation and cytokine expression in HIV-1-associated neurologic diseases. Adv Neuroimmunol. 1995;5:335–58. 8748077 10.1016/0960-5428(95)00012-q

[148] Zamani N, Osgoei LT, Aliaghaei A, Zamani N, Hassanian-Moghaddam H. Chronic exposure to methadone induces activated microglia and astrocyte and cell death in the cerebellum of adult male rats. Metab Brain Dis. 2023;38:323–38. 36287354 10.1007/s11011-022-01108-z

[149] Zeis T, Enz L, Schaeren-Wiemers N. The immunomodulatory oligodendrocyte. Brain Res. 2016;1641:139–48. 26423932 10.1016/j.brainres.2015.09.021

[150] Zhang SZ, Wang QQ, Yang QQ, Gu HY, Yin YQ, Li YD, Hou JC, Chen R, Sun QQ, Sun YF, Hu G, Zhou JW. NG2 glia regulate brain innate immunity via TGF-β2/TGFBR2 axis. BMC Med. 2019;17:204. 31727112 10.1186/s12916-019-1439-x PMC6857135

[151] Zhuang JL, Wang CY, Zhou MH, Duan KZ, Mei YA. TGF-β1 enhances Kv2.1 potassium channel protein expression and promotes maturation of cerebellar granule neurons. J Cell Physiol. 2012;227:297–307. 21412780 10.1002/jcp.22735

