# Correction: The Neuroimmune System and the Cerebellum

**DOI:** 10.1007/s12311-025-01859-2

**Published:** 2025-05-23

**Authors:** Donna L. Gruol

**Affiliations:** Neuroscience Department, The Scripps Research Institute, La Jolla, CA 92037 USA

**Correction: The Cerebellum (2023) 23:2511-2537**

10.1007/s12311-023-01624-3

In the original version of the article, the legend of Figure 11 inaccurately cited the source of the published image. The correct citation is “Reprinted with modifications from “Microglia-triggered plasticity of intrinsic excitability modulates psychomotor behaviors in acute cerebellar inflammation” by Yamamoto, M., Kim, M., Imai, H., Itakura, Y., and Ohtsuki, G., 2019, Cell Rep, 28 [146]).

The original article has been corrected.

